# POH1 deubiquitylates and stabilizes E2F1 to promote tumour formation

**DOI:** 10.1038/ncomms9704

**Published:** 2015-10-29

**Authors:** Boshi Wang, Aihui Ma, Li Zhang, Wei-Lin Jin, Yu Qian, Guiqin Xu, Bijun Qiu, Zhaojuan Yang, Yun Liu, Qiang Xia, Yongzhong Liu

**Affiliations:** 1 State Key laboratory of Oncogenes and Related Genes, Shanghai Cancer Institute, Renji Hospital, Shanghai Jiaotong University School of Medicine, Shanghai 200032, China; 2 Institute of Nano Biomedicine and Engineering, Department of Instrument Science and Engineering, Key Laboratory for Thin Film and Microfabrication Technology of Ministry of Education, School of Electronic Information and Electronic Engineering, Shanghai Jiaotong University, Shanghai 200240, China; 3 Department of Liver Surgery, Renji Hospital, Shanghai Jiaotong University School of Medicine, Shanghai 200217, China

## Abstract

Hyperactivation of the transcriptional factor E2F1 occurs frequently in human cancers and contributes to malignant progression. E2F1 activity is regulated by proteolysis mediated by the ubiquitin–proteasome system. However, the deubiquitylase that controls E2F1 ubiquitylation and stability remains undefined. Here we demonstrate that the deubiquitylase POH1 stabilizes E2F1 protein through binding to and deubiquitylating E2F1. Conditional knockout of *Poh1* alleles results in reduced E2F1 expression in primary mouse liver cells. The POH1-mediated regulation of E2F1 expression strengthens E2F1-downstream prosurvival signals, including upregulation of Survivin and FOXM1 protein levels, and efficiently facilitates tumour growth of liver cancer cells in nude mice. Importantly, human hepatocellular carcinomas (HCCs) recapitulate POH1 regulation of E2F1 expression, as nuclear abundance of POH1 is increased in HCCs and correlates with E2F1 overexpression and tumour growth. Thus, our study suggests that the hyperactivated POH1–E2F1 regulation may contribute to the development of liver cancer.

## Abstract (web summary)

The transcription factor E2F1 controls the expression of multiple genes and is frequently overactivated in cancer. Here, the authors show that E2F1 is deubiquitinated by POH1 and that this enhances the role of E2F1 in cell survival, and contributes to the pathogenesis of liver cancer.

## Graphical abstract

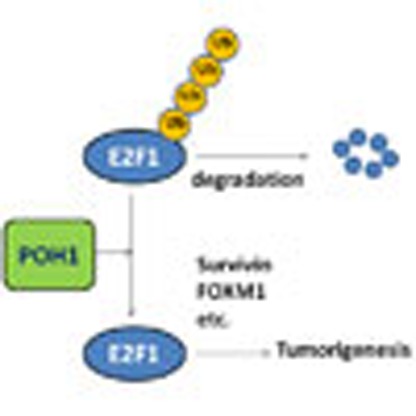

The E2F transcription factor 1 (E2F1) is a master transcription factor that participates in numerous important biological processes^1^. Besides the clinical evidence that aberrant upregulation of E2F1 frequently occurs in various types of human cancer and correlates with malignant progression and poor survival prognosis^2^,^3^,^4^, the E2F1-driven onocgenic activity has been reinforced in different models based on E2F1 transgenic or knockout mice^5^,^6^,^7^,^8^. Although the observations showing that E2F1 is involved in cellular senescence and apoptotic response may suggest its dual role in tumorigenesis^9^, several events contributing to tumorigenesis may counterbalance the tumour suppressive effects of E2F1. For instance, cells with the deficiencies in p53 or p14(ARF) can escape from E2F1-mediated apoptosis or prosenescent effects^6^,^10^, and the protumorigenic signals generated by epidermal growth factor receptor and phosphatidylinositol 3-kinase (PI3K)/Akt are capable of inhibiting E2F1 apoptotic function^11^,^12^. In addition, E2F1 itself has been shown to promote tumour cell survival and EMT as well as angiogenesis in certain circumstances^13^,^14^,^15^,^16^. Therefore, the oncogenic activity of E2F1 is determined by the strength of prosurvival factors either downstream of this transcriptional factor or provided by other signals.

E2F1 turnover is controlled by the ubiquitin–proteasome system^17^,^18^,^19^,^20^,^21^. Several factors responsible for the ubiquitination of E2F1 have been identified, including SKP2, APC/C and the ROC–cullin complex^17^,^18^,^19^,^21^. MDM2, which negatively regulates p53, directly interacts with and increases the half-life of E2F1 protein by displacing SCF^20^. Deubiquitination is considered a key process in the maintenance of proper cellular function and homeostasis. Numerous studies have established that the dysfunction of deubiquitinating enzymes is critical for tumour development, progression or chemosensitivity^22^,^23^,^24^,^25^,^26^. However, the contribution of deubiquitinating enzymes to the stabilization of E2F1 and its biological significance in carcinogenesis has not been determined.

POH1/rpn11/PSMD14 is a deubiquitinating enzyme within the 19S particle lid that regulates proteasomal activities^27^,^28^. POH1 plays a ‘proof-reading' role in controlling the fate of incoming substrates^27^,^28^,^29^. In mammalian cells, POH1 functions in various biological processes, including double-strand DNA break responses^30^, embryonic stem cell differentiation^31^, aggresome disassembly and clearance^32^, cellular viability^33^,^34^, multidrug resistance^35^ and protein stability^36^,^37^,^38^. However, whether POH1 deregulation occurs in and contributes to the development of liver cancer has not been determined.

In this study, we identify POH1 as the deubiquitinating enzyme that stabilizes E2F1 and demonstrate that aberrant hyperactivity of POH1–E2F1 regulation promotes liver tumour formation. Our study therefore describes a previously unknown mechanism by which E2F1 expression is regulated as well as its implication in tumorigenesis.

## Results

### Identification of POH1 as a positive regulator of E2F1

**Figure 1:**
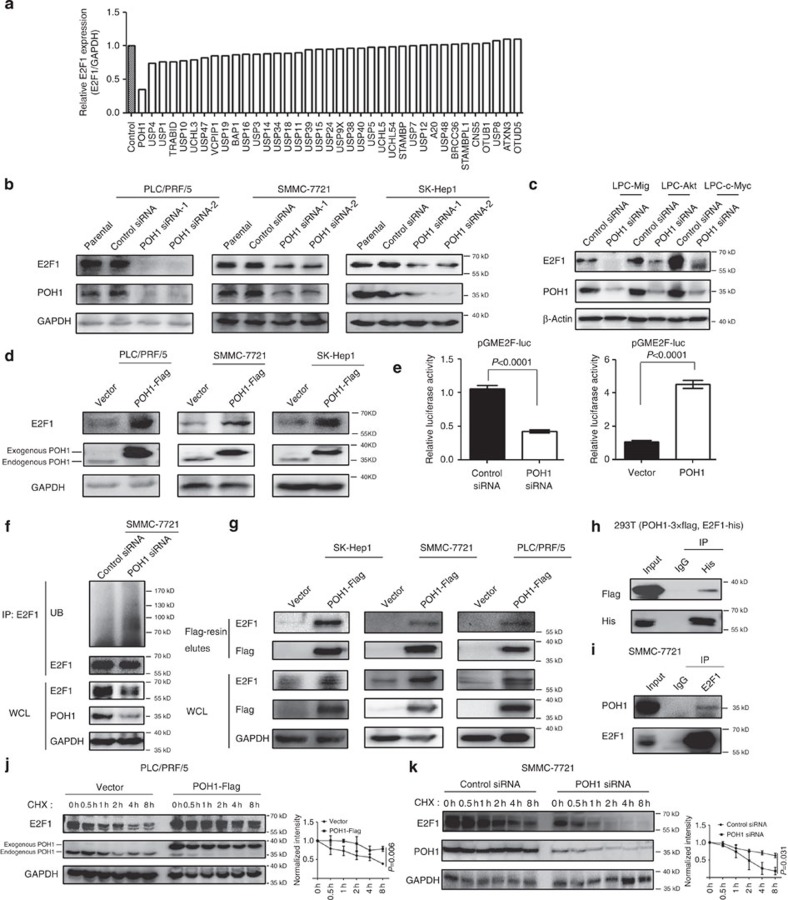
POH1 is a positive regulator of E2F1 stability. (**a**) For each DUB protein indicated, a pool of three independent siRNAs was transfected into PLC/PRF/5 cells. The protein levels of E2F1 were analysed by immunoblotting and normalized to that of the control GAPDH. The relative expression compared with that of the siRNA control is presented in rank order. (**b**,**c**) Immunoblot analysis of POH1 and E2F1 in human liver tumour cells (**b**) and mouse LPCs (**c**) transfected with the indicated siRNAs. Untransfected cells were shown as parental controls. (**d**) Immunoblot analysis of POH1 and E2F1 in human liver tumour cells with or without POH1-Flag expression. (**e**) Luciferase assay in HEK293T cells co-transfected with the reporter plasmids combined with the indicated siRNAs (left panel) or with POH1 and the vector control, respectively (right panel). Data are mean±s.d. (by *t-*test analysis, *P* values are shown in the graph, *n*=3). (**f**) SMMC-7721 cells transfected with control siRNA or POH1 siRNA were cultured for 72 h. Cell lysates were immunoprecipitated with anti-E2F1 antibody, and the immunocomplexes were immunoblotted with antibodies against UB and E2F1. (**g**) Human liver tumour cells transfected with POH1-Flag were immunoprecipitated with Flag-M2 agarose beads. The eluates were immunoblotted for the detection of E2F1. (**h**) HEK293T cells were co-transfected with E2F1-His and POH1-Flag followed by immunoprecipitation using anti-His antibody or IgG. The immunocomplexes were analysed by immunoblotting. (**i**) Endogenous E2F1 proteins were immunoprecipitated with anti-E2F1 antibody or IgG, and then analysed by immunoblotting. (**j**) PLC/PRF/5 cells with or without stable expression of POH1-Flag were treated with CHX (100 μg ml^−1^) for the indicated time points. The cell lysates were examined by immunoblotting (left panel). A plot of normalized amount of E2F1 protein is shown (right panel). (**k**) SMMC-7721 cells were transfected with either control siRNA or POH1 siRNA for 48 h, followed by CHX(100 μg ml^−1^) treatment for the indicated times. The cell extracts were analysed by immunoblotting (left panel). A plot of normalized amount of E2F1 protein is shown (right panel). Data in panels (**j**,**k**) are mean±s.d. (by ANOVA analysis, *P* values are shown in the graphs, *n*=3).

To identify deubiquitinating enzymes (DUBs) with the capacity of regulating E2F1 expression, we initially screened 37 DUBs, expression of which are relatively high in human liver tissues based on in sillico EST profile analysis. For each DUB tested we employed a pool of three non-overlapping siRNA oligos for transfection experiments. The results of the relative quantification of E2F1 protein levels, as assembled in rank order, showed that knockdown of POH1 markedly repressed E2F1 expression (Fig. 1a). Representative images of the immunoblots are shown in Supplementary Fig. 1a. The mRNA levels of POH1 were increased in a number of hepatocellular carcinomas (HCCs) compared with that in the adjacent non-tumour specimens (Supplementary Fig. 1b), suggesting a potential clinical relevance for POH1 in the development of HCC.

We further validated the downregulation of E2F1 expression by POH1 knowdown in different liver cancer cell lines (Fig. 1b; Supplementary Fig. 1c). The decrease in E2F1 protein was not associated with changes in the E2F1 mRNA levels in SMMC-7721 and PLC/PRF/5 cells (Supplementary Fig. 1d), indicating that a post-transcriptional mechanism is involved in POH1-mediated regulation of E2F1. Furthermore, in *p53*^−/−^ mouse liver progenitor cells transformed with myristoylated AKT (LPC-Akt) or c-Myc (LPC-c-Myc), and the control cells (LPC-Mig)^39^, POH1 depletion markedly inhibited E2F1 expression (Fig. 1c; Supplementary Fig. 1e). The effects of POH1 on E2F1 expression might also apply to other types of cancer cells such as the colon cancer LoVo cells (Supplementary Fig. 1f). Remarkably, the expression levels of E2F2 and E2F3, two E2F1 relatives^9^, were not significantly reduced (Supplementary Fig. 1g), indicating that POH1-mediated regulation of E2F1 may be a selective event.

We examined the effect of gain of POH1 function on E2F1 activity in tumour cells. PLC/PRF/5, SMMC-7721 and SK-Hep1 cells stably expressing exogenous Flag-tagged POH1 had higher levels of E2F1 than did the control cells (Fig. 1d; Supplementary Fig. 1h). POH1 overexpression positively regulated E2F1 activity, as monitored by an E2F1-binding element-luciferase reporter, whereas POH1 deletion yielded an opposite effect (Fig. 1e). More importantly, we did not observe an appreciable change in proteasomal activity within the cells overexpressing POH1, as revealed by the levels of total ubiquitinated proteins (Supplementary Fig. 1i). These results indicate that POH1-mediated upregulation of E2F1 is not paralleled by a change in the global activity of the proteasome.

### POH1 stabilizes E2F1

To investigate whether POH1 regulates E2F1 ubiquitination, we first examined the levels of polyubiquitin-modified E2F1 in tumour cell lines with or without POH1 knockdown. POH1 knockdown significantly enhanced the levels of E2F1 ubiquitination (Fig. 1f). This finding led us to hypothesize that POH1 may interact with and deubiquitinate E2F1. We therefore performed immunoprecipitation analyses, and the results revealed that E2F1 was co-immunoprecipitated with POH1 protein in cells transfected with Flag-tagged POH1 (Fig. 1g). The interaction between POH1 and E2F1 was verified by co-immunoprecipitation assays in HEK293T cells (Fig. 1h). Furthermore, the interaction between endogenous POH1 and E2F1 was demonstrated by co-immunoprecipitation (Fig. 1i). We then generated a set of truncated mutants of POH1 and E2F1 to delineate the regions within these proteins essential for their interaction. Depletion of the COON-terminal domain (aa181–310) of POH1 significantly impaired the association between E2F1 and POH1, whereas the portion of E2F1 (aa201–437) displayed strong capability to interact with POH1 (supplementary Fig. 2a,b). The interaction between the N-terminal fragment (aa1–200) of E2F1 and POH1 was relatively weak, but certainly reproducible. To test whether POH1 stabilizes E2F1 protein, we examined the effect of both POH1 depletion and overexpression on the stability of endogenous E2F1 protein in the presence of the inhibitor of protein translation, cycloheximide (CHX). The results indicate that forced POH1 expression significantly potentiates E2F1 protein stability (Fig. 1j). In contrast, E2F1 protein was degraded more rapidly in POH1-knockdown cells compared with control cells (Fig. 1k). These data collectively suggest that POH1 interacts with and stabilizes E2F1 in tumour cells.

### E2F1 downregulation in mouse liver cells deficient in *Poh1*

**Figure 2:**
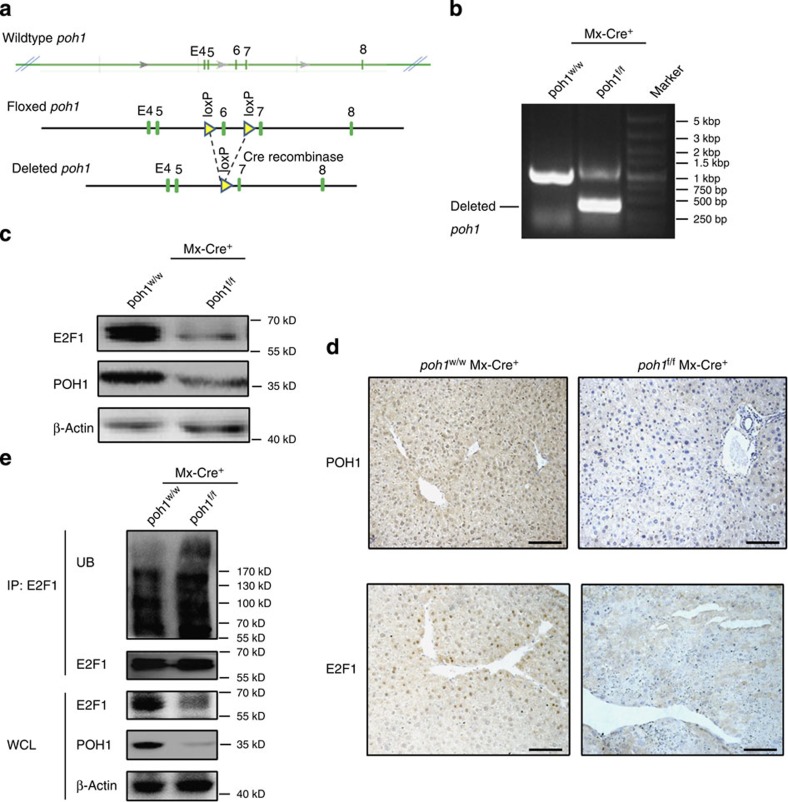
POH1 depletion in primary liver cells decreases E2F1 protein levels. (**a**) Schematic diagram of the wild-type and the floxed *poh1* alleles. Mice with a floxed *poh1* allele were crossed with a Cre line to generate deleted *poh1* allele. (**b**) PCR analysis of genomic DNA demonstrating that the floxed *poh1* alleles are efficiently deleted in Mx-Cre^+^ and *poh1*^f/f^ mouse liver tissues. (**c**) POH1 and E2F1 immunoblotting in liver tissues from *poh1*^w/w^, Mx-Cre^+^ and *poh1*^f/f^, Mx-Cre^+^ mice treated with three rounds of polyI:C injections. (**d**) IHC analysis of POH1 and E2F1 expression in liver tissues. Representative images are presented. Scale Bar, 100 μm. (**e**) Cell lysates of mouse liver tissues with (*poh1*^f/f^, Mx-Cre^+^) or without (*poh1*^w/w^, Mx-Cre^+^) POH1 deletion were immunoprecipitated with anti-E2F1 antibody, and the immunocomplexes were immunoblotted with indicated antibodies.

To evaluate whether POH1-mediated regulation of E2F1 occurs in primary mouse liver cells deficient in POH1, we generated conditional *Poh1*^f/f^ mice by targeting *Poh1* exon 6 (Fig. 2a), the deletion of which led to loss of POH1 expression in the presence of Cre recombinase. The inducible *Poh1* knockout mice (Mx-Cre^+^, *Poh1*^f/f^), generated by crossing *Poh1*^f/f^ mice with Mx-Cre mice, were treated with three rounds of polyinosinic:polycytidylic acid (polyI:C) injection. Disruption of the floxed *Poh1* allele was then achieved in liver tissues and accompanied by a substantial reduction of E2F1 expression (Fig. 2b,c). Immunochemistry staining verified the reduction in both POH1 and E2F1 expression in mouse hepatocytes (Fig. 2d). Consistent with results obtained in cultured cells, the levels of ubiquitin-modified E2F1 were substantially increased in the liver tissues of mice with *poh1* ablation (Fig. 2e). Accordingly, depletion of *Poh1* in *poh1*^f/f^ MEFs could cause an acceleration of E2F1 protein turnover that apparently was not due to the change in E2F1 mRNA levels (Supplementary Fig. 3a–c).

### POH1 regulates E2F1 polyubiquitination

**Figure 3:**
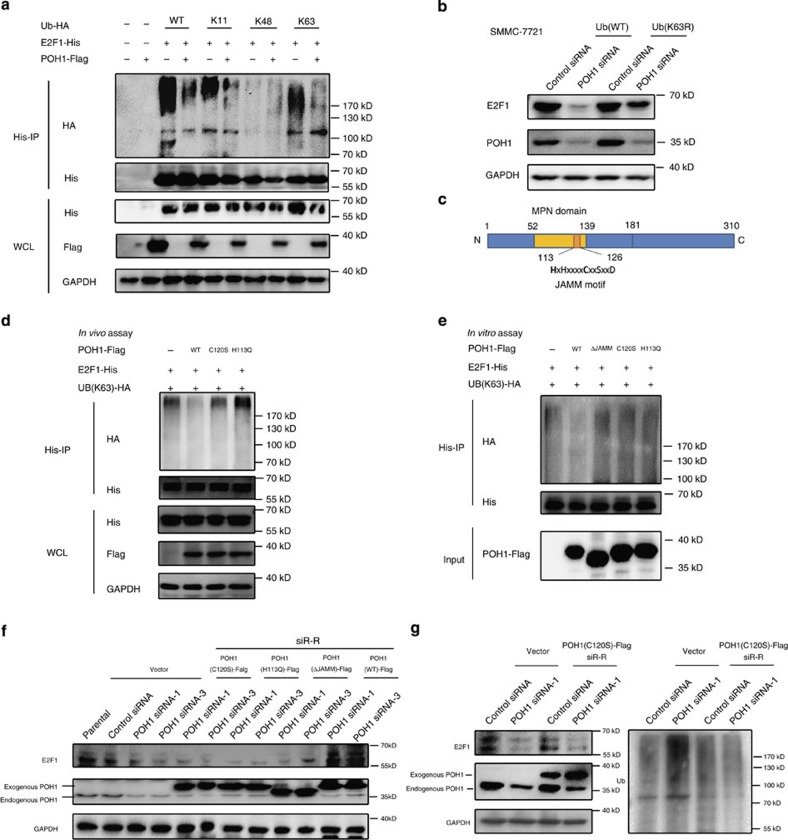
POH1 reverses K63- and K11-linked polyubiquitination of E2F1. (**a**) HEK293T cells overexpressing E2F1-His, POH1-Flag, and HA-tagged, different forms of ubiquitin were treated with MG132 (10 μM) for 6 h. Cell lysates were immunoprecipitated using anti-His antibody. Ubiquitination levels were detected using anti-HA antibody. (**b**) SMMC-7721 cells transfected with UB(WT) or UB(K63R) were cultured for 72 h in the presence of control siRNA or POH1 siRNA. Cell lysates were analysed by immunoblotting using the indicated antibodies. (**c**) A schematic diagram of the JAMM motif within the MPN domain of POH1. (**d**) HEK293T cells were co-transfected with E2F1-His, vector, or the POH1-Flag, and UB (K63)-HA constructs. Cell lysates were immunoprecipitated with anti-His antibody and immunoblotted with the indicated antibodies. (**e**) UB (K63)-HA modified E2F1-His proteins were purified from HEK293T cells with protein G beads and anti-His antibody. The protein complexes containing POH1-Flag or the Flag-tagged C120S or H113Q mutants of POH1 were purified from HEK293T cells with anti-Flag M2 beads. The purified UB (K63)-HA-E2F1 and POH1-Flag protein complexes were incubated for 1 h in the reaction buffer. After the reaction, E2F1-His proteins were further purified with anti-His antibody and immunoblotted with antibodies against HA and His. (**f**) SMMC-7721 cells were transfected with control siRNA or POH1 siRNA together with the siRNA-resistant (siR-R) forms of Flag-tagged WT or mutant POH1. The cells were collected 72 h after transfection and immunoblotted with the indicated antibodies. (**g**) SMMC-7721 cells stably expressing the siRNA-resistant POH1 (C120S)-Flag were transfected with control siRNA or POH1 siRNA and cultured for 72 h.The cell lysates were analysed by immunoblotting to determine the levels of E2F1 and total ubiquitin-modified proteins.

We next determined which types of polyubiquitin modifications on E2F1 protein were affected by POH1-mediated deubiquitnation. E2F1 proteins in the lysates of cells transfected with E2F1-His, POH1-Flag, and each one of the different ubiquitins (WT, K11-, K48-, or K63-only ubiquitin-HA), separately, were purified and subjected to immunoblotting analysis using an anti-HA antibody. POH1 overexpression substantially reduced K11- and K63-linked ubiquitination on E2F1 (Fig. 3a). Similar results were obtained in the experiments using the antibodies recognizing K48 or K63-linked polyubiquitin chains (Supplementary Fig. 4a,b). The cleavage of K63-linked polyubiquitin chains on E2F1 by POH1 is consistent with the function of POH1 described as a K63-specific deubiquitinase in previous structural and biochemical studies^40^,^41^. Of note, K11-linked ubiquitination is related to E2F1 degradation by the proteasomal^19^. Overexpression of the K63-resistant form of ubiquitin (K63R) attenuated POH1 knockdown-induced E2F1 downregulation, indicating that K63-linked polyubiquitination is important for POH1-mediated E2F1 turnover (Fig. 3b). Although POH1 overexpression caused the deubiqutination of E2F1, the approach did not have an appreciable impact on levels of total polyubiquitin-modified proteins (Supplementary Fig. 5a), suggesting that POH1-mediated deubiquitination of E2F1 can take place in cells in absence of a noticeable alteration in global proteasome activity. In addition, in MG132-treated cells, wherein the global proteasomal activity was inhibited, ectopic POH1 expression was able to deubiquitinate E2F1 (Supplementary Fig. 5b), suggesting that this effect is independent of the overall proteasome function.

Previous structural and functional studies indicate that the JAMM domain of POH1 and the C120 and H113 sites are important for the deubiquitinating enzymic activity of POH1 (Fig. 3c)^36^,^37^,^42^,^43^. Accordingly, ectopic expression of the POH1 mutants C120S or H113Q failed to deubiquitinate of E2F1 proteins (Fig. 3d). Of note, the POH1 mutant with deletion of JAMM motif (deletion of 113–126aa) was capable of binding to E2F1 (Supplementary Fig. 2a). Our *in vitro* analysis using 293T cell-derived recombinant POH1 further confirmed the capability of POH1 for cleaving K63-linked polyubiquitin chains from E2F1 and the importance of the functional domain and sites (Fig. 3e). Unexpectedly, E.coli-expressed POH1 did not display a deubiquitining activity on E2F1; that was consistent with the previous studies^28^,^36^, suggesting that the defect might be due to either inappropriate folding of prokaryotic recombinant POH1 or absence of POH1-partner proteins in reaction. Furthermore, we generated different siRNA-resistant (siR-R) POH1 constructs for rescue assays, and the results showed that restoration of POH1 expression with the C120S-, H113Q- and ΔJAMM-POH1 mutants did not attenuate the inhibition of E2F1 caused by the deletion of endogenous POH1 (Fig. 3f). Altogether, we have demonstrated that the deubiquitinating activity that POH1 possesses is fundamental for POH1 regulation of E2F1.

It should be noted that POH1 may function separately in regulating E2F1 abundance and proteasomal activities. C120S-POH1 was enzymatically dead and not effective in deubiquitinating E2F1 (Fig. 3d,e). As expected, ectopic expression of the siRNA-resistant C120S-POH1 did not restore E2F1 expression in cells where the endogenous POH1 was downregulated by siRNA (Fig. 3g left). However, C120S-POH1 expression is sufficient in maintaining the global proteasomal activities, as revealed by the abundance of ubiquitin-modified proteins (Fig. 3g right). These results are consistent with the findings that the C120 mutation has a deleterious effect on POH1 deubiquitinating activities but does not abolish its proteasome activator function^31^,^44^. Altogether, these results indicate that POH1-mediated deubiquitination of E2F1 may be independent of the canonical function of POH1 in the proteasome.

### POH1 has protumorigenic activities

**Figure 4:**
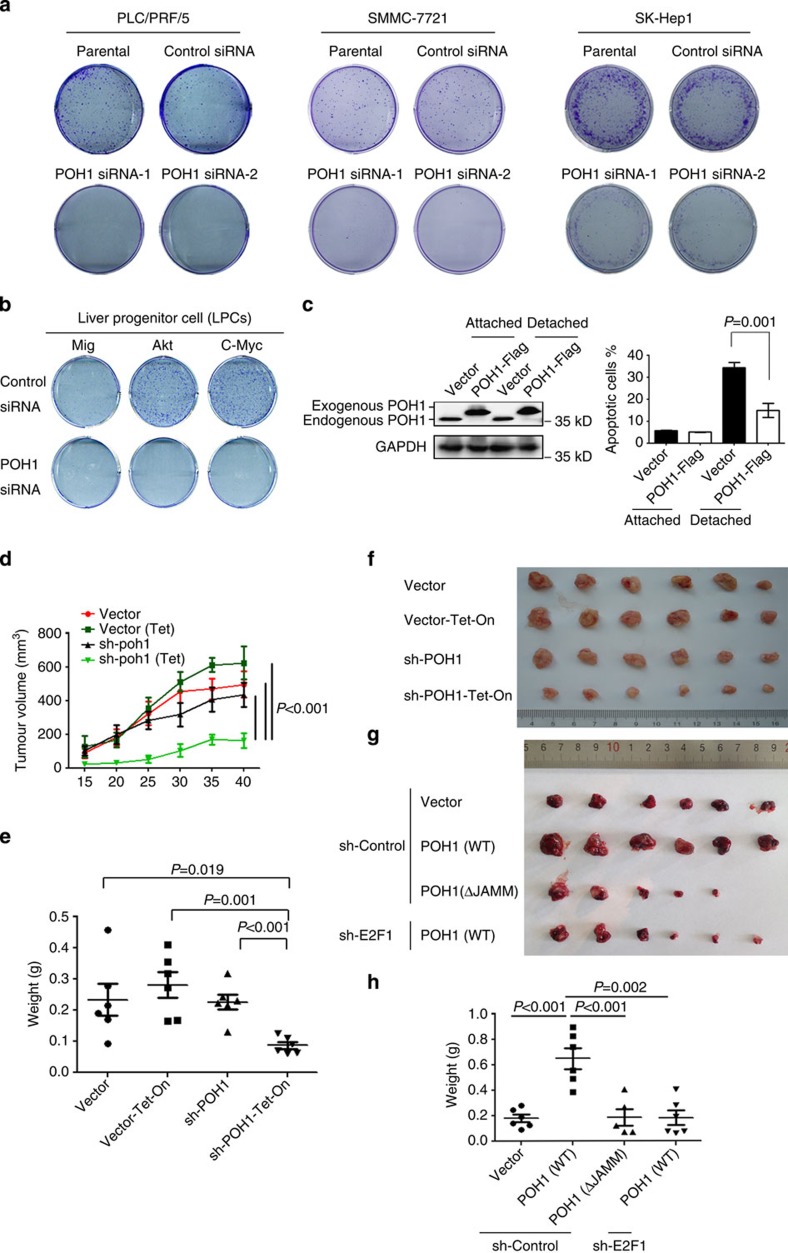
POH1 inhibition suppresses tumour cell growth *in vitro* and *in vivo*. (**a**) Human liver tumour cells with or without transfection of control siRNA or POH1 siRNA were examined by colony-formation assay. The representative results of three independent experiments are presented. (**b**) Mouse LPC-Mig, LPC-Akt and LPC-c-Myc cells transfected with control siRNA or POH1 siRNA were cultured for the colony-formation assays. The representative results of three independent experiments are presented. (**c**) PLC/PRF/5 cells with or without stable expression of POH1-Flag were cultured in attached or detached conditions for 72 h. Cells were subjected to apoptosis analysis. Data shown are mean±s.d. (by *t-*test, *P* value is shown in the graph, *n*=3). (**d**) SK-Hep1 cells with an inducible Tet-On-sh-POH1 expression cassette (sh-POH1) and control cells (empty vector) were subcutaneously injected into the right flanks of male BALB/c nude mice. The mice of each group were then randomly divided into two sub-groups and provided with normal drinking water (*n*=6) or with water containing 2 mg ml^−1^ doxycycline (Tet-On) (*n*=6). The graph indicates tumour growth in the mice at the end of the experiment. Tumour volumes were measured at the indicated time intervals. Data shown are mean±s.e.m. *P* values calculated by ANOVA test are shown in the graph. (**e**) The tumour weights were quantified. Data shown are mean ±s.e.m. *P* values calculated by *t*-test are shown in the graph. (**f**) A photograph of the tumours in each group is presented. (**g**,**h**) Tumour formation in male BALB/c nude mice (*n*=5 or 6) injected subcutaneously with PLC/PRF/5 cell lines as indicated, the photograph of the tumours in each group is shown in (**g**); the tumour weight was quantified in (**h**). Data shown are mean ±s.e.m. *P* values calculated by *t*-test are shown in the graphs.

We next investigated the effects of POH1 inhibition on the malignant growth potential of liver cancer cells. POH1 inhibition by siRNAs markedly suppressed the colony-forming ability of three human liver cancer cell lines and the oncogenes-transformed mouse LPCs and the control cells (Fig. 4a,b; Supplementary Fig. 6a). Consistently, MTT assays also revealed reduced viability of human liver cancer cells with POH1 knockdown (Supplementary Fig. 6b). In addition, POH1 knockdown resulted in increased percentage of apoptotic cells (Supplementary Fig. 6c–e) and cell cycle arrest (Supplementary Fig. 6f). Conversely, ectopic POH1 expression substantially potentiated tumour cell survival in detached culture conditions (Fig. 4c). Ectopic expression of C120S-POH1, the enzyme-dead mutant with the capacity of maintaining overall proteasomal activity, did not rescue the suppression of cell proliferation caused by endogenous POH1 deletion (Supplementary Fig. 6g,h).

To address the importance of POH1 in regulating the growth of liver cancer cells, we generated the SK-Hep1 cells with a Tet-On inducible shRNA cassette against POH1 (sh-POH1-Tet-on; Supplementary Fig. 6i).Compared with the control groups, mice with induced sh-POH1 expression in tumour cells displayed a significant inhibition of tumour growth (*P*<0.001, by analysis of variance (ANOVA) analysis) (Fig. 4d–f). Immunohistochemical (IHC) staining revealed reduced E2F1 and Ki67 expression in the xenograft tumours generated from POH1-knockdown cells (Supplementary Fig. 7). On the contrary, POH1 but not the JAMM-deleted POH1 overexpression in the PLC/PRF/5 liver cancer cells significantly increased the tumours' volumes (Fig. 4g,h). Furthermore, E2F1 knockdown with stable shRNA expression was able to counteract the protumorigenic activities of POH1 (Fig. 4g,h). These results underscore an essential role of E2F1 in POH1-mediated protumorigenic activities.

### POH1 activates Survivin and FOXM1 expression through E2F1

**Figure 5:**
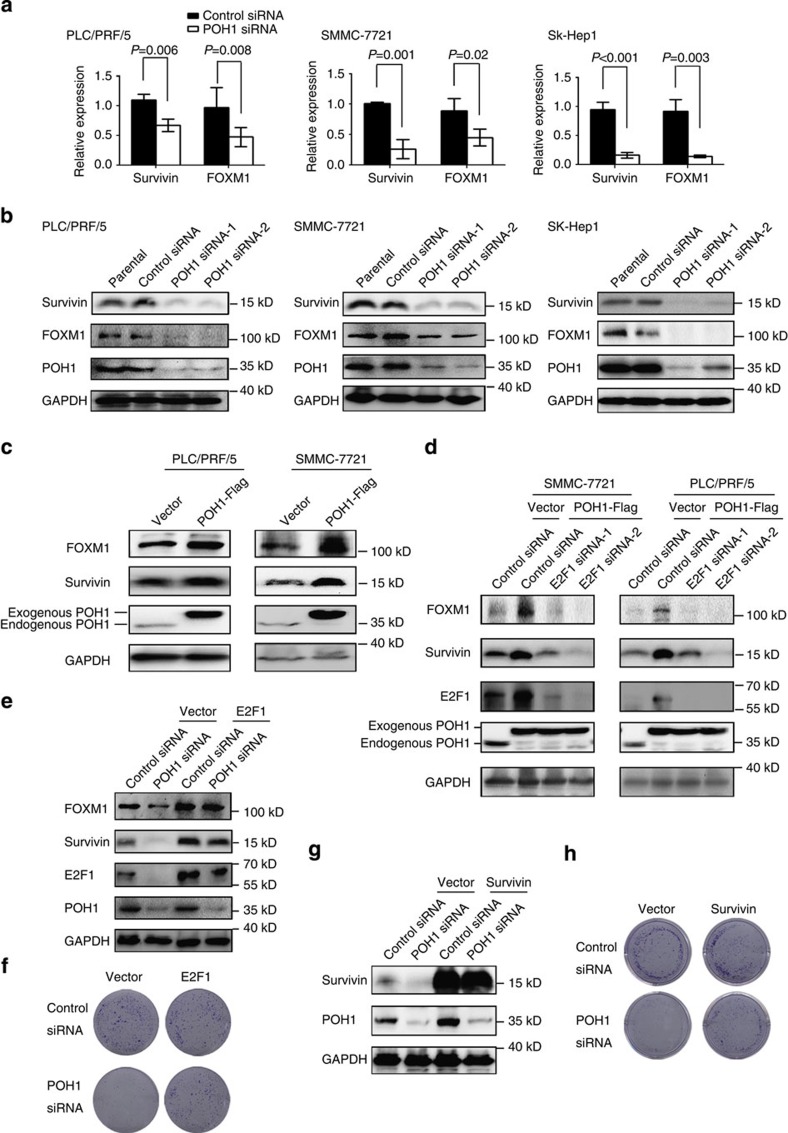
POH1 increases Survivin and FOXM1 expression by regulating E2F1. (**a**) Real-time RT–PCR analysis of the mRNA levels of Survivin and FOXM1 in cells transfected with control siRNA or POH1 siRNA. Data are mean±s.d. (by *t-*test analysis, *P* values are shown in the graph, *n*=3) (**b**) Human liver tumour cells untransfected (parental) or transfected with control siRNA or POH1 siRNA-1 and -2 were harvested at 72 h. The cell lysates were analysed using the indicated antibodies. (**c**) PLC/PRF/5 and SMMC-7721 cells transduced with lentivirus containing POH1-Flag and the control were analysed by immunoblotting using the indicated antibodies. (**d**) Cells overexpressing POH1-Flag or control cells were transfected with control siRNA or siRNA against E2F1 for 48 h. Cell lysates were analysed by immunoblotting using the indicated antibodies. (**e**,**f**) PLC/PRF/5 cells overexpressing E2F1 were transfected with control siRNA or POH1 siRNA, 72 h after transfection, cell lysates were analysed by immunoblotting (**e**); cells were cultured for 10 days then stained with crystal violet. Representative data from the colony-formation assays are presented, *n*=3 (**f**). (**g**,**h**) PLC/PRF/5 cells overexpressing Survivin were transfected with control siRNA or POH1 siRNA, and cell lysates were analysed by immunoblotting (**g**); representative data from the colony-formation assays are presented, *n*=3 (**h**).

To delineate the roles of POH1 in regulating tumour cell survival and proliferation, we examined genome-wide transcription profiles of HCC SMMC-7721 cells with or without POH1 knockdown by mRNA microarray. To identify the downstream effectors of the POH1-E2F1 pathway, we focused on the expression alterations of a set of known E2F1 target genes between the control and the POH1-depleted cells (Supplementary Fig. 8 and Supplementary Table 1). The results showed that the majority of E2F1 targets examined were downregulated in the POH1-depleted cells, including Survivin and FOXM1, both of which are critical for tumorigenesis^45^,^46^,^47^,^48^. The downregulation of Survivin and FOXM1 in the cells with POH1 deletion was validated by real-time RT–PCR (Fig. 5a). Accordingly, POH1 deletion substantially inhibited the expression of Survivin and FOXM1 proteins in human liver cancer cell lines (Fig. 5b; Supplementary Fig. 9a). Conversely, the forced expression of exogenous POH1 significantly elevated Survivin and FOXM1 levels (Fig. 5c; Supplementary Fig. 9b), whereas the overrexpression of an enzymatic dead mutant of POH1 failed to up-regulate Survivin and FOXM1 (Supplementary Fig. 10). Consistent with these findings, E2F1 knockdown robustly inhibited Survivin and FOXM1 expression and increased the frequency of apoptosis in SMMC-7721 cells (Supplementary Fig. 11a,b). Furthermore, E2F1 inhibition by siRNAs almost entirely abolished POH1-mediated upregulation of Survivin and FOXM1 in liver cancer cells (Fig. 5d). The restoration of E2F1 or Survivin expression in liver cancer cells substantially counteracted POH1 deletion-induced cell growth inhibition and apoptotic response (Fig. 5e–h; Supplementary Fig. 12). Altogether, these results have demonstrated that E2F1 is responsible for POH1- mediated activation of the protumorigenic factors.

### Nuclear POH1 is essential for liver tumour cell growth

**Figure 6:**
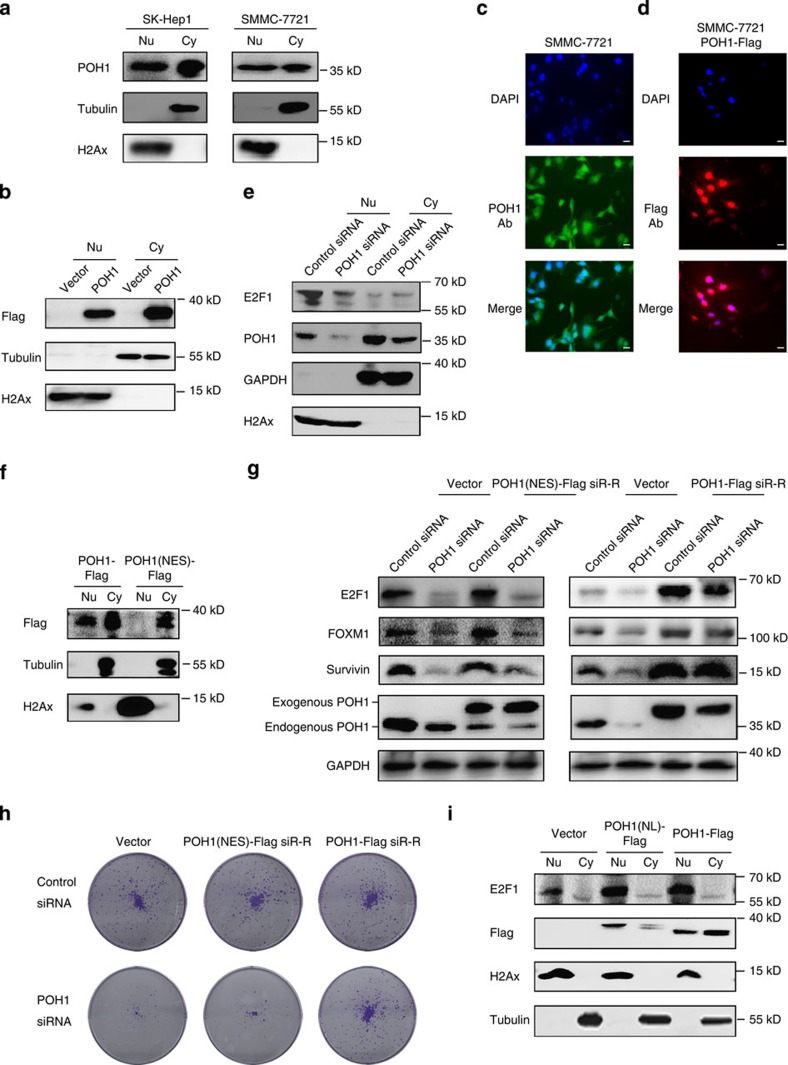
The cytoplasm-restricted expression of POH1 fails to increase E2F1-Survivin/FOXM1 expression. (**a**) The nuclear (Nu) and cytoplasmic (Cy) fractions of tumour cells were analysed with antibodies against POH1, histone H2Ax (the indicative for nuclear fraction) and tubulin (a control for the cytoplasmic fraction). (**b**) The nuclear and cytoplasmic fractions of the SMMC-7721 cells stably expressing POH1-Flag were analysed using anti-Flag antibody. (**c**,**d**) Fluorescent staining of SMMC-7721 cells and the SMMC-7721 cells stably expressing POH1-Flag using anti-POH1(**c**) and anti-Flag antibodies (**d**), respectively. Nuclear DNA was visualized using DAPI staining. Scale bar, 20 μm. (**e**) The nuclear (Nu) and cytoplasmic (Cy) fractions of SMMC-7721 cells transfected with either control or the POH1 siRNA were extracted and analysed using the indicated antibodies. (**f**,**g**) SMMC-7721 cells stably expressing the siRNA-resistant forms of POH1-Flag or of POH1(NES)-Flag were analysed by immunoblotting using the indicated antibodies (**f**); cells were transfected with control or POH1 siRNA for 72 h, and the cell extracts were then analysed by immunoblotting (**g**). (**h**) SMMC-7721 cells expressing two different forms of POH1 were transfected with control or POH1 siRNA. 48 h after transfection, the cells were cultured for 10 days. The cells were stained with crystal violet. (**i**) The nuclear (Nu) and cytoplasmic (Cy) fractions of SMMC-7721 cells transfected with control or the nuclear-localized POH1. The cell extracts were then analysed by immunoblotting.

Given that POH1 may function in the nucleus^30^,^37^,^49^, we hypothesized that the nuclear localization of POH1 is critical for regulating E2F1 expression. We first isolated the nuclear and cytoplasmic fractions of SK-Hep1 and SMMC-7721 cells and measured the expression pattern of POH1. Indeed, both endogenous and exogenous POH1 proteins were detected in the nuclear and cytoplasmic compartments with antibodies against POH1 and Flag-tag, respectively (Fig. 6a,b). We further substantiated the observation by fluorescent immunostaining (Fig. 6c,d). In cells with POH1 knockdown, E2F1 expression in the nuclear fractions was substantially decreased (Fig. 6e). E2F1 protein was mainly localized in tumour cell nucleus and the co-immunoprecipitation between POH1 and E2F1 was primarily observed in the purified nuclear compartment of the cells (Supplementary Fig. 13). To investigate the biological function of nuclear POH1, we incorporated a nuclear export signal (NES) sequence into the NH_2_ terminus of POH1-Flag. The POH1(NES) is prominently located in the cytoplasmic compartment (Fig. 6f). We therefore comparatively analysed the effectiveness of the siRNA-resistant forms of POH1 and POH1(NES) on restoring the levels of E2F1, Survivin and FOXM1 in cells with endogenous POH1 knockdown. In contrast with POH1 that entirely reinstated the expression of these proteins, POH1(NES) did not compensate for the loss of function of the endogenous POH1 (Fig. 6g). More importantly, cytoplasm-restricted expression of POH1 did not alleviate the inhibition of cell growth and the induction of cell apoptosis caused by endogenous POH1 deletion (Fig. 6h and Supplementary Fig. 14a,b). In addition, the POH1 mutant protein (POH1(NL)-Flag) with a predominant distribution in cell nucleus efficiently upregulated the expression of E2F1 protein (Fig. 6i). Collectively, these data demonstrate that nuclear-localized POH1 is critical for E2F1 stabilization and the efficient growth of liver tumour cells.

### POH1 is upregulated in HCCs and correlates with E2F1 levels

**Figure 7:**
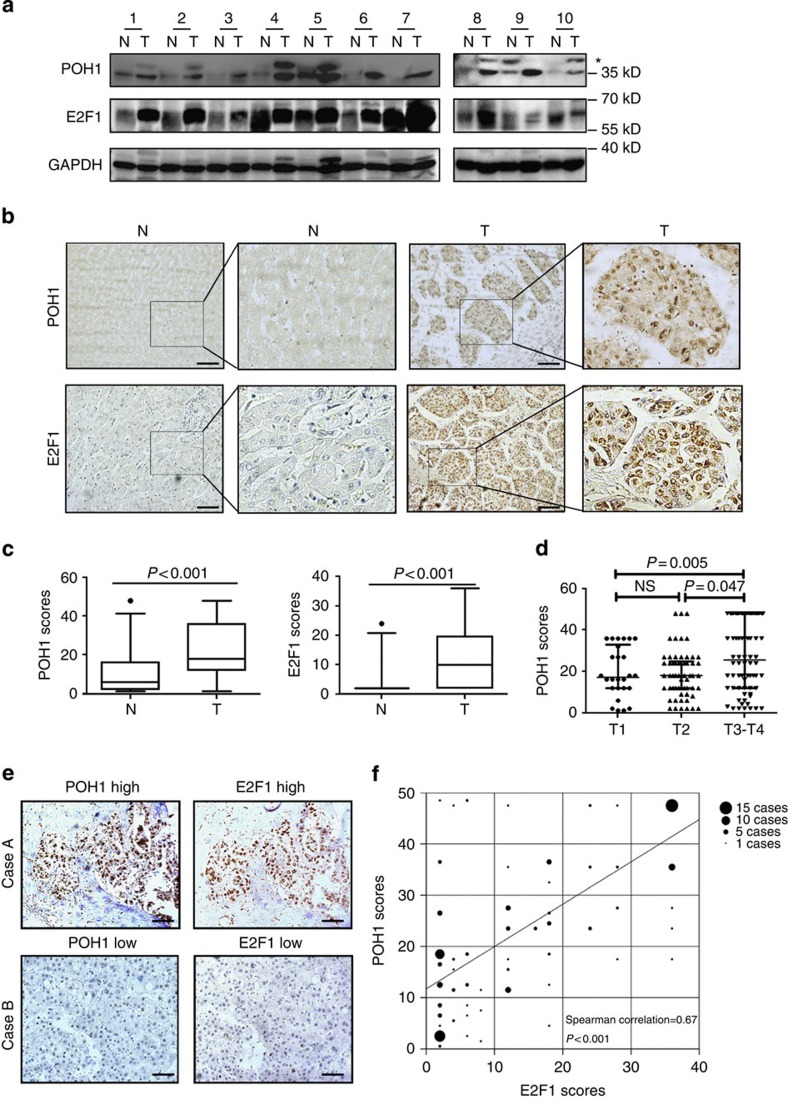
POH1 expression is upregulated in human HCC tissues and correlates with E2F1 abundance. (**a**) Immunoblotting analysis of POH1 and E2F1 expression in 10 pairs of HCCs and non-tumoral liver tissues. *Non-specific band. (**b**) POH1 immunochemical staining in 154 pairs of HCCs and non-tumoral tissues; representative photographs are presented. Scale bar, 100 μm. (**c**) The expression scores of POH1 and E2F1 for HCCs and non-tumoral liver tissues. Box-and-whisker plots are shown Boxes represent the upper and lower quartiles and median; whiskers show the data points that are neither lower than the first percentile nor greater than the 99th percentile. *P* values calculated by Wilcoxon signed-rank test are shown. (**d**) Correlation between POH1 scores and tumour stages in HCC samples. The scatter dot plot and the median with interquartile range are presented. *P* values from the multiple hypothesis testing (Holm–Sidak's multiple comparisons test) are shown. (**e**) Case A: a representative specimen with high POH1 and E2F1 staining. Case B: a representative specimen with low POH1 and E2F1 staining. Scale bar, 100 μm. (**f**) Correlation between POH1 and E2F1 expression in HCC samples are shown (by Spearman correlation test). All *P* values are shown in the graphs.

To further delineate whether POH1-mediated regulation of E2F1 expression is clinically relevant to human HCC development, we characterized the expression patterns of POH1 and E2F1 and assessed their association in human HCC samples. Western blot analyses showed elevated POH1 and E2F1 expression in 10 HCCs in comparison with their adjacent non-tumoral tissues (Fig. 7a). To examine POH1 and E2F1 expression in a relatively large pool of HCC samples, we employed tissue microarrays containing 154 matched HCCs and the adjacent non-malignant liver tissues for IHC staining. Cytoplasmic immunoreactivity was observed in both hepatocellular carcinoma cells and normal hepatocytes. In total, 41.6% (64/154) of the non-tumour liver tissues exhibited clear nuclear staining, whereas in the HCCs, 72.1% (111/154) of the samples displayed nuclear staining (Fig. 7b, Supplementary Fig. 15a). IHC staining for POH1 and E2F1 was scored according to the intensity (ranging from 1 to 4) and the percentage of positive cells. Given that nuclear POH1 mainly contributed to E2F1 regulation, the staining location was also added to the scores. The overall score was then determined by multiplying the intensity score, the percentage score, and the location score (Supplementary Fig. 15b). The increases in the expression of POH1 and E2F1 in HCCs were statistically significant (Fig. 7c). Moreover, we detected a significant correlation between POH1 staining scores and tumour stages of the samples examined with Kruskal–Wallis test (*P=*0.015). In addition, the multiple hypothesis test (Holm–Sidak's multiple comparisons test) was performed and these results showed that the protein levels of POH1 in stage T3/T4 tumours were significantly higher than those in tumours at earlier stages (T1 versus T2, *P*=0.845; T1 versus T3–T4, *P*=0.047; T2 versus T3–T4, *P*=0.005) (Fig. 7d). However, there was no correlation between POH1 levels with tumour grades (Supplementary Fig. 16). Importantly, a positive correlation was found between the staining scores of POH1 and E2F1 (*P*<0.001, by Spearman correlation test; Fig. 7e,f). Thus, these results reveal the clinical relevance of POH1-mediated regulation of E2F1 in HCC development.

**Figure 8:**
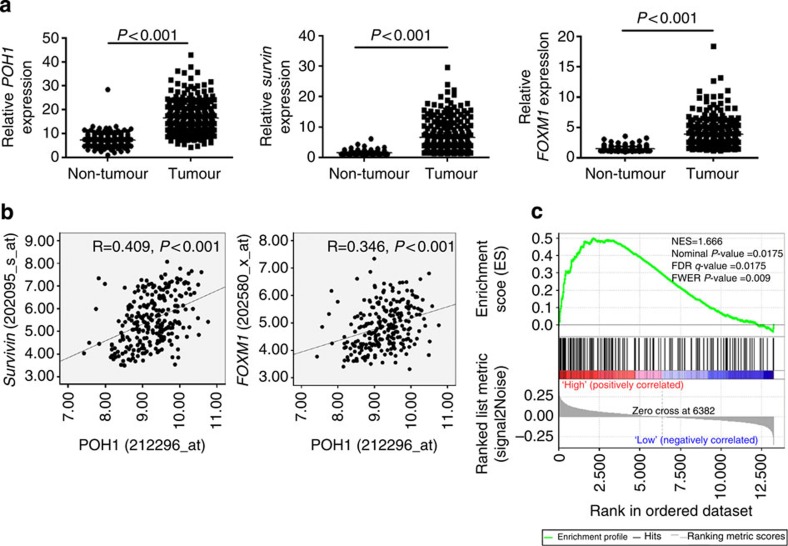
Correlation of POH1 expression with E2F1 target gene signature in HCC dataset. (**a**) Transcript levels of *POH1*, *Survivin* and *FOXM1* in non-tumoral liver and HCC tissues from the GSE14520 data set. The expression levels of these genes were compared using *t*-test. (**b**) The expression of *POH1* correlates with *Survivin* and *FOXM1* levels. The Pearson correlation analyses were performed. (**c**) Gene set enrichment analysis of the HCC dataset GSE14520 based on a signature of E2F1 target genes and POH1 expression.

To further corroborate the findings aforementioned, we analysed a publicly available transcriptome data set (GSE14520), which was collected from 241 non-tumoral liver tissues and 247 HCC samples. The transcript levels of POH1 and its downstream genes Survivin and FOXM1 in the tumour tissues were aberrantly upregulated in comparison with those in the non-tumour samples (Fig. 8a). Moreover, the POH1 transcript abundance was positively correlated with the expression of Survivin and FOXM1 in the HCC samples (Survivin: Pearson correlation *R*=0.409, *P*<0.001; FOXM1: Pearson correlation *R*=0.346, *P*<0.001, Fig. 8b). To assess the correlation between POH1 expression and E2F1 target gene signature, we performed the gene set enrichment analysis (GSEA) of the dataset GSE14520 using a signature of E2F1-target genes from the Molecular Signature Database (http://www.broadinstitute.org/gsea/msigdb/cards/E2F1_UP.V1_UP.)^50^,^51^. The positive enrichment score obtained from the analyses with the nominal *P* value=0.0175, false discovery rate *Q* value=0.0175 and FWER *P* value=0.009 (Fig. 8c), is indicative of a high enrichment in the expression of E2F1 target gene signature in the tumour subset with high POH1 expression.

## Discussion

Emerging evidence indicates that POH1 plays roles in several biological processes, including DNA repair, cell differentiation and transcriptional control. Our present study provides evidence that POH1 deubiquitinates and stabilizes the master transcription factor E2F1 and functions as a tumour-promoting protein in HCCs. We demonstrated that POH1 efficiently deubiquitinates E2F1 by removing the K-63 polyubiquitin chains, and this observation is consistent with previous studies revealing that the JAMM domain within POH1 is responsible for removing K63-linked ubiquitin chains^40^,^41^,^43^. The deubiquitination of E2F1-K63 polyubiquitin chains appears to be important for POH1-mediated stabilization of E2F1 because Ub-K63R overexpression showed a dominant-negative effect that rescued E2F1 expression in cells with POH1 inhibition (Fig. 3b). Although protein modification via K63-linked polyubiquitin chains is not generally considered a degradation signal, previous studies have suggested that K63-linked polyubiquitin chains serve as proteasome recognition tags by which substrates are efficiently processed in proteasome^52^,^53^. Of note, the proteasome indeed degrades K63-Ub chain-modified substrates^54^. The modification of K11-linked polyubiquitination on E2F1 is substantially downregulated by POH1 (Fig. 3a). The K11-linked polyubiquitin chains are involved in the proteasomal degradation of E2F1 protein^19^, raising the possibility that loss of the K11-linked polyubiquitin modification may contribute to POH1-mediated stabilization of E2F1. In addition, the possibility cannot be excluded that mutual regulation between K11- and K63-modification on E2F1 may occur; if so, then the deubiquitination of the K63-modification by POH1 may cause a reduction in K11-linked polyubiquitination of E2F1 proteins. Furthermore, the formation of mixed K11/K63 chains on E2F1 may also need to be taken into consideration. Of note, although E2F1 deubiquitination by POH1 has been revealed to be important for E2F1 stabilization, the molecular details of the regulation remain to be defined. An approach using point mutants of these proteins that specifically disrupt the E2F1/POH1 interaction may deepen our understanding of POH regulation of E2F1. Designing such mutants is currently difficult owing to lack of structural insights into the interaction, but warrants our future studies.

The 19S regulatory particle localized in the nucleus may display non-proteolytic functions through directly binding the transcriptional regulatory elements in various conditions^55^. In primary liver tissues and cultured cell lines, we observed a considerable amount of POH1 in the nucleus. The existence of nuclear POH1 is important for regulating E2F1 because cytoplasmic-localized POH1 cannot stabilize E2F1 in cells with deletion of endogenous POH1. We propose that the deubiquitination of E2F1 by POH1 primarily occurs in the nucleus; as a consequence, the prosurvival genes, including Survivin and FOXM1, are transcriptionally activated. A previous study indicated that POH1 overexpression in HEK293 cells downregulates c-Jun ubiquitination, resulting in c-Jun accumulation and upregulation of AP1-mediated gene expression^36^. These observations suggest that transcriptional regulation by the 19S proteasome is not simply due to direct binding to and sequential activation of the targeted locus.Instead, deubiquitinating enzymes of the 19S particle may provide an important alternative by deubiquitinating the master transcriptional factors. Therefore, these findings shed light on the mechanism by which the 19S particle or its subunits play transcriptional regulatory roles in the nucleus.

Most importantly, our study has revealed a coordinated upregulation of POH1 and E2F1 expression in clinical HCC samples, implying a pathological significance for POH1 regulation of E2F1 in HCC development. E2F1 is thought to play dual roles in tumorigenesis^9^, and E2F1 downstream targets consist of both oncogenes and tumour suppressors. The paradoxical function of E2F1 is cell context dependent and determined by the output and nature of downstream targets, which are either beneficial or deleterious to cell proliferation and survival^12^,^56^. Some oncogenic factors activated by E2F1, including Akt, mTORC1 and EGR1, can override the tumour-suppressive networks downstream of E2F1, producing a growth advantage to the tumour cells^12^,^13^,^57^. Consistently, we demonstrated that the anti-apoptotic factors Survivin and FOXM1 are downstream of the POH1-E2F1 axis. Importantly, these prosurvival factors are involved in the development of various types of cancer, including liver cancer^47^,^48^. The results based on data mining reveal that the abundance of POH1 positively correlates with the expression of Survivin and FOXM1 in HCC tissues and demonstrate the activation of E2F1 target genes in the subset with high POH1 expression. In summary, we propose that the aberrant upregulation of nuclear POH1-mediated E2F1 stabilization is an important event in the development of liver cancer and that targeting POH1 might serve as a promising strategy for cancer treatment.

## Methods

### Cell lines and tissue specimens

PLC/PRF/5, SK-Hep1 and HEK293T cells were acquired from the American Type Culture Collection (ATCC, Manassas VA, USA). Authentication of these cell lines was performed using the GenePrint10 System (Promega Biotech Co.) and via comparisons to the ATCC STR database. All the STR profiles yielded 100% matches. The SMMC-7721 cell line was obtained from the cell bank of the Institute of Biochemistry and Cell Biology of the Chinese Academy of Sciences (Shanghai, China). Mouse LPC-Mig, LPC-Akt, and LPC-c-Myc cells were generated in our laboratory^39^. Primary *poh1*^w/w^ and *poh1*^f/f^ MEFs were obtained from embryos at embryonic day 13.5 from *poh1*^w/w^ and *poh1*^f/f^ mice. The HEK293T cells and the packaging plasmids were used for lentivirus production. Transduced cells were isolated by puromycin selection or FASC sorting. Cell lines were maintained at 37 °C in 5% CO_2_ in DMEM supplemented with 10% fetal bovine serum. Cell lines were tested routinely for mycoplasma before use in an experiment. Human HCC samples and matched non-tumoral liver tissues were obtained from the Renji Hospital, Shanghai Jiaotong University, China. The use of human material was approved by the ethical review committee of Renji Hospital and informed consent was obtained from these subjects. Three sets of commercially available tissue microarrays (TMA) containing 154 HCC and non-tumoral tissue pairs were used for IHC staining.

### Reagents and primary antibodies

Doxycycline (DOX), CHX and puromycin were from Sigma Aldrich. MG132 were from Millipore. Lipofectamine 2000 or Lipofectamine 3000 Transfection Reagent was from Invitrogen. The following antibodies used for western blotting: POH1 (Proteintech, 12059-1-AP, 1:1,000), E2F3 (Proteintech, 12334-1-AP, 1:1,000), FOXM1 (Proteintech, 13147-1-AP, 1:500), E2F1(C-20) (Santa Cruz, sc-193, 1:500), E2F1(KH95) (Santa Cruz, sc-251, 1:500), E2F2 (Santa Cruz, sc-632, 1:500), GAPDH (Santa Cruz, sc-25778, 1:1,000), β-actin (Santa Cruz, sc-47778, 1:1,000), α-Tubulin (Santa Cruz, sc-69969, 1:1,000), Survivin (Cell Signaling Technology, 2808, 1:1,000), ubiquitin (abcam, EPR8589, 1:1,000), H2Ax (abcam, EPR895, 1:1000), HA-tag (Sigma Aldrich, H9658, 1:1,000), Flag-tag (Sigma Aldrich, f1804, 1:1,000), His-tag (MBL, D291-3, 1:1,000), 6 × His tag (HuaAn, M0812-3, 1:1,000), anti-Ubiquitin Antibody, Lys48-Specific (Millipore, 05-1307, 1:500) and anti-Ubiquitin Antibody, Lys63-Specific (Millipore, 05–1313, 1:500). The following antibodies and reagents for immunoprecipitation included 6 × His tag (Cell Signaling Technology, 2366, 1:100), His tag (MBL, PM032, 1:100), E2F1 (Santa Cruz;sc-193 or sc-251, 1:100), the Flag M2 Affinity Gel (Sigma Aldrich, A2220). The antibodies for immunofluorescence were POH1 (Sigma Aldrich, HPA002114, 1:200) and Flag (Sigma Aldrich, f1804, 1:200). The following antibodies were used for immunohistochemistry: POH1 (Sigma Aldrich, HPA002114, 1:500), E2F1 (Santa Cruz, sc-251, 1:25), Ki67 (abcam, EPR3611, 1:500). The adenovirus expressing Crerecombinase was purchased from HANBIO. Inc.

### Plasmids and siRNAs

Flag (3 × Flag)-tagged POH1, POH1 mutants (the ΔJAMM, C120S and H113Q mutant), POH1 with the depletion of carboxyl terminal (181–310aa) and the NH2 terminal (1–180aa) mutant their siRNA-resistant forms, as well as His-tagged E2F1 (full length and a set of truncated mutants) and Survivin were cloned into pLVX (Clontech 632187). The Flag-tagged POH1 (NES) and POH1(NL) were also cloned into pLVX vector. The NES was incorporated at the NH_2_ terminus of POH1 and the nuclear localized signal (NLS) was added at both the NH_2_ and carboxyl terminus of POH1, along with the mutation of the putative NES (212–221aa) LEQKMLLNL within POH1 to AEQKMAANA. A POH1 shRNA expression sequence was cloned into a Tet-On expression vector ptripz (Open Biosystems) by the replacement of the original expressing ORFs. A shE2F1 expression sequence was cloned into PPRIME plasmid. For the luciferase reporter assay, the pGME2F-luc plasmid was purchased from Genomeditech. The duplex siRNAs were chemically synthesized by Genepharma (Shanghai, China), and the sequences of the siRNAs were as follows: POH1 si-1 (targeting human and mouse POH1, 5′-GGTCTTAGGACATGAACCA-3′), POH1 si-2 (5′-GTGATTGATGTGTTTGCTA-3′), h-POH1 si-3 (targeting 3′-UTR in human POH1 mRNA, 5′-CAGTCTCAGTTGTGCAATT-3′) and h-E2F1 (si-1: 5′-GTGATTTATTTATTGGGAA-3′; si-2: 5′-CACTGAATCTGACCACCAA-3′). The plasmids expressing HA-tagged wild-type ubiquitin (Addgene plasmid 17608), K63-ubiquitin (Addgene plasmid 17606) and K48-ubiquitin (Addgene plasmid 17605) were kindly provided by Ted Dawson. The plasmid expressing HA-tagged K11-ubiquitin (Addgene plasmid 22901) were kindly provided by Sandra Weller. The plasmid expressing K63R-ubiquitin were kindly provided by Dr Xiaoren Zhang (SIBS, CAS).

### Co-immunoprecipitation

Cells were collected and lysed with an IP lysis buffer (Beyotime Institute of Biotechnology, P0013). Total protein (up to 5 mg) was incubated with 50 μl of Protein G-agarose suspension (Millipore, 16–266,) for 3 h at 4 °C on a rocking platform to reduce non-specific binding. After removing the beads, the supernatant was supplemented with the primary antibodies followed by incubation for an additional 3 h at 4 °C. A total of 100 μl of Protein G-agarose was then added to each immunoprecipitation mixture, and the incubation was continued overnight at 4 °C on a rocking platform. The immunoprecipitates were collected by centrifugation and washed three times with the cold 1 × TBS. After the loading buffer was added, the agarose was boiled and subjected to western blot analysis. The EasyBlot anti-mouse (GTX221667-01) or EasyBlot anti-rabbit (GTX221666-01) IgG HRP-conjugated secondary antibodies (Genetex) were employed to avoid the denatured heavy and light chains from antibodies used in immunoprecipitation assays.

The Flag M2 Affinity Gel (Sigma, A2220) was used to immunoprecipited the Flag-tagged proteins, After three times' washes with cold 1 × TBS the immunoprecipited protein complexes were eluted through 3 × Flag peptides (Sigma, F4799). The co-immunoprecipated proteins were detected through western blot assay.

### Immunohistochemistry

In the immunocytochemical assay, the slides were rehydrated and immersed in 3% hydrogen peroxide solution for 15 min; pretreated by microwave for 25 min in 0.01 mol l^−1^ citrate buffer, pH 6.0, at 95 °C; and cooled for 60 min at room temperature. In between each incubation step, the sections were washed with PBS, pH 7.4. The slides were blocked by 10% normal goat serum for 30 min at 37 °C, washed, and then incubated overnight at 4 °C with diluted antibody against each protein studied. After washing with PBS, the slides were visualized using GTVisionTMIII Detection System/Mo&Rb (GeneTech, GK500710) following the manufacturer's instructions. IHC staining for POH1 and E2F1 was cored according to the intensity (1: low staining; 2: moderate staining; 3: high staining; 4: extremely high staining), the percentage of positive cells (1: 0–25%; 2: 26–50%; 3: 51–75%; 4: 76–100%), and the location of staining (1: no nuclear staining or the intensity of nuclear staining was weaker than that of cytoplasm; 2: the intensity of nuclear staining was equal to that of cytoplasm; 3: the intensity of nuclear staining was higher than that of cytoplasm). The overall score=intensity score × percentage score × location score.

### Xenograft model

The right flanks of male BALB/c nude mice (5 weeks of age) were subcutaneously injected with SK-Hep1 cells (5 × 10^6^ in 0.2 ml PBS) infected with virus expressing either ptripz-sh-POH1or empty vector (*n*=12 per group). Each group of mice was then randomly divided into two groups (*n*=6 per group). One group was administered doxycycline (2 mg ml^−1^) to induce to the POH1 shRNA expression and the other group was provided normal water. Tumour growth was monitored every 5 days. The tumour-bearing mice were sacrificed 40 days after inoculation, and the tumours were removed for further study (photographing, weighing, fixing and paraffin-embedding). The left and right flanks of male BALB/c nude mice (5 weeks of age) were subcutaneously injected with PLC/PRF/5 cells (6 × 10^6^ in 0.2 ml PBS). The tumour-bearing mice were killed 35 days after inoculation. All experiments were subject to approval by the Animal Care and Use Committee of Shanghai Cancer Institute.

### Quantitative real-time RT–PCR

Total cellular RNA from tissues and cells were extracted by the RNAiso Plus kit (Takara Bio Inc.) and cDNA preparation was preformed according to standard procedures using primeScript RT Master kit (Takara Bio Inc.). Real-time RT–PCR was performed by SYBR gGreen quantitative PCR kit (Takara Bio Inc.) using the 7300 Real-Time PCR System or ViiA7 System (AB Applied Biosystems). The primers used in the mRNA levels detection were as follows: human POH1-F: 5′-TTGCTATGCCACAGTCAGGA-3′, human POH1-R: 5′-AACAACCATCTCCGGCCTTC-3′; human GAPDH-F: 5′-CATGAGAAGTATGACAACAGCCT-3′, human GAPDH-R: 5′-AGTCCTTCCACGATACCAAAGT-3′; human E2F1-F: 5′-AGCGGCGCATCTATGACATC-3′, human E2F1-R: 5′-GTCAACCCCTCAAGCCGTC-3′; human Survivin-F: 5′-AGCCAGATGACGACCCCAT-3′, human Surviving-R: 5′-TGGCTCTTTCTCTGTCCAGT-3′; human FOXM1-F: 5′-ACGTCCCCAAGCCAGGCTC-3′, human FOXM1-R: 5′-CTACTGTAGCTCAGGAATAA-3′. Mouse E2F1-F: 5′-CAACTGCAGGAGAGTGAGCA-3′, Mouse E2F1-R: 5′-GTCCTGGCAGGTCACATAGG-3′; Mouse GAPDH-F: 5′-AGGTCGGTGTGAACGGATTTG-3′, Mouse GAPDH-R: 5′-TGTAGACCATGTAGTTGAGGTCA-3′.

### Generation of *Poh*1 conditional knockout mice

*Poh1* knockout mouse model was created by Beijing Biocytogen. In brief, homology regions covering 8.0 kb upstream of *Poh1* exon 6 and 5.7 kb downstream of exon 6 were subcloned from a BAC clone (RP23-1O11; Invitrogen) from C57BL/6J mouse genomic BAC library. FRT-flanked Neo resistance-positive selection cassette was inserted downstream of exon 6 and two loxP sites were introduced upstream of exon 6 and downstream of exon 6, respectively. After linearization, the targeting vector was transfected into C57BL/6J embryonic stem cells (Biocytogen) by electroporation. Seven positive clones were identified by Southern blotting with 5′-probe, 3′-probe and Neo probe. Two positive clones were injected into Balb/c blastocysts and implanted into pseudopregnant females. Chimeric mice were crossed with C57BL/6J mice to obtain F1 mice carrying the recombined allele containing the floxed *Poh1* allele and Neo selection cassette. These mice were mated with Flp recombinase expressing C57BL/6J Flp mice to remove the Neo resistance cassette and generate a line of Neo-excised floxed mice. Heterozygous Neo-excised, *Poh1*-floxed mice were then crossed with C57BL/6J Mx-Cre mice to achieve the genomic deletion of *Poh1* exon 6 from the floxed alleles. Pathogen-free mice were housed under specific pathogen-free conditions and fed food and water regularly. All of the experiments involving mice were performed according to the Institutional Animal Care and Use Committee of Shanghai and the National Research Council Guide for Care and Use of Laboratory Animals. Genotyping was performed by PCR using the proper primers. By crossing Mx-Cre mice with *Poh*1^f/f^ mice and then backcrossing, we generated interferon-inducible *Poh1* knockout mice. *Poh*1^f/f^, Mx-Cre^+^ mice (6–8 weeks old) were injected with a total of three injections of 5 μg g^−1^ body weight of polyI:C to induce *Poh1* deletion. The *Poh1* WT, Mx-Cre^+^ mice (6–8 weeks old) were administered the same amount of polyI:C injections on the same dosing schedule; these mice were used as controls. The primers used to detect the deleted *poh1* include F: 5′-TGTGATTGCTGTTTTATGAGGCA-3′ and R: 5′-GAGAATGACAACTATTGGGAGACTTAGC-3′.

### Western blot

Cells or tissues were lysed with RIPA buffer (Thermo Fisher Scientific, 89901) containing protease inhibitors cocktail (Roche Diagnostics, 05892970001) and phosphatase inhibitor cocktail (Roche Diagnostics, 04906845001). The lysates were clarified by centrifugation at 13,000 *g* for 30 min at 4 °C. The total protein concentration was estimated using a BCA protein assay kit (Thermo Fisher Scientific, 23225). Protein samples (50–150 μg) were loaded on to and separated using SDS/PAGE, transferred on to NC membranes (Pall Corporation) blocked and probed with the primary antibodies. After washing, the blots were incubated with goat anti-rabbit (Santa Cruz, sc-2004) or goat anti-mouse (Santa Cruz, sc-2005) HRP (horseradish peroxidase)-conjugated secondary antibodies (Santa Cruz Biotechnology) and visualized using the SuperSignal West Dura Extended Duration Substrate (Thermo Fisher Scientific, 34076). The uncropped versions of western blots are shown in Supplementary Fig. 17.

### *In vivo* deubiquitination assay

HA-ubiquitinated 6 × His- E2F1 or endogenous E2F1 was immunoprecipited using the anti-6 × His-tag antibodies (CST) or E2F1 antibodies (santa Cruz), respectively, in denaturing conditions. The E2F1-His protein was purified and immunoblotted with antibodies against HA or ubiquitin.

### *In vitro* deubiquitination assay

UB(K63)-HA conjugated E2F1-His was purified from HEK293T cells with protein G-agarose (Millipore, 16–266) incubated with anti-His antibody. The protein complexes containing Flag tagged wild-type POH1 or the C120S, H113Q mutant type of POH1 were purified from HEK293T cells with anti-Flag M2 Affinity Gel (Sigma) followed by eluted through 3 × Flag peptides (Sigma). UB(K63)-HA-E2F1 and the POH1 protein complexes were incubated for 1 h, at 37 °C in the reaction buffer (50 mM Tris PH7.5, 10 mM MgCl2, 1 mM DTT, 100 mM NaCl, 1 mM ATP). After reaction, the E2F1-His protein was purified and immunoblotted with antibodies against HA.

### Immunofluorescence staining

Cells were seeded on coverslips and washed with PBS buffer. Cells were fixed with 4% paraformaldehyde at room temperature for 15 min and permeabilized with 0.1% Triton X-100 for 10 min. After washing with PBS buffer, cells were incubated with a blocking solution (5% goat serum) for 30 min at room temperature and then incubated with primary antibodies overnight at 4 °C. Cells were then washed three times and incubated with the anti-mouse or anti-rabbit Alexa Fluor secondary antibodies (dilution 1:200, Invitrogen) for 30 min at room temperature. DNA was counterstained using 4, 6 -diamidino-2-phenylinodole (DAPI). The slides were observed under a ZEISS Axiovert 200 fluorescence optics microscope (Zeiss Shanghai, China).

### Cell viability analysis

Cells with different treatments were seeded at 1,500 cells in 200μl DMEM per well in 96-well culture plates. At the indicated time points, 20 μl of 0.5 mg ml^−1^ MTT (Thiazolyl Blue Tetrazolium Bromide, M5655, Sigma) was added to each well and incubated at 37 °C for 3 h. Then, the culture was replaced with 150 μl dimethyl sulphoxide (D8418, Sigma) to stop the reaction. The absorbance values (OD 590 nm) were measured using a spectrophotometer (Thermo Fisher Scientific).

### Colony-formation assays

To assay the proliferation potential of cells, cells were seeded at 2,000 cells in 2 ml DMEM per well in the six-well culture plates. After 10 days' culture, cells were fixed with methanol and stained with crystal violet (Beyotime, C0121).

### Apoptosis analysis

For apoptosis analysis, cells were placed in six-well plates with the confluence of 50% 24 h before analysis, cells were harvested by trypsin digestion, washed with binding buffer and stained with 5 μl annexin-V-PE (BD, 556422) or annexin-V-APC (ebioscience, BMS306APC-100) and 0.5 μl 7-AAD (7-aminoactinomycin D, A9400, Sigma) in 100 μl binding buffer for 30 min at room temperature in the dark. Apoptotic cells were detected by flow cytometry using a FACSCalibur flow cytometer (BD). For anoikis analysis, cells were cultured in the 6-well plates with polystyrene-coated low attachment surface (3471, Corning Incorporated) for 72 h, then cells were collected for annexin V/7-AAD double staining.

### Cell cycle analysis

Cells were placed in six-well plates with the confluence of 50% 24 h before analysis; cells were collected by trypsin digestion, and resuspended in 75% ethanol at 4 °C overnight. The cells were collected by centrifugation and washed with 1 × PBS. Finally, the cells were resuspended in 1 × PBS containing 100 g ml^−1^ RNase A and 50 g ml^−1^ propidium iodide (PI). After incubation for 30 min at 37 °C in dark, samples were subjected to flow cytometry for cell cycle analysis.

### Luciferase reporter assay

HEK293T cells were transiently transfected with luciferase reporter plasmid (300 ng), POH1 expression plasmid (1 μg) or POH1 siRNA (100 nM). To correct the transfection efficiency variations in each group of transfection, 20 ng of the Renilla luciferase plasmids were co-transfected in each experiment. Forty-eight hours post-transfection, the firefly and Renilla luciferase activities were monitored using the Dual-Luciferase Reporter Assay System (Promega). The data are shown as the ratio of firefly to Renilla luciferase activity.

### Microarray analysis

Total RNA was extracted using TRIZOL Reagent (Cat#15596-018, Life technologies) following the manufacturer's instructions and checked for a RINnumber to inspect RNA integrity by an Agilent Bioanalyzer 2100 (Agilent technologies). Qualified total RNA was further purified by RNeasy micro kit (Cat#74004, QIAGEN, GmBH, Germany) and RNase-Free DNase Set (Cat#79254, QIAGEN). Total RNA were amplified, labelled and purified by using GeneChip 3′-IVT ExpressKit (Cat#901229, Affymetrix) followed the manufacturer's instructions to obtain biotin labelled cRNA. Array hybridization and wash was performed using GeneChip Hybridization, Wash and Stain Kit (Cat#900720, Affymetrix) in Hybridization Oven 645 (Cat#00-0331-220V, Affymetrix, Santa Clara, CA, USA) and Fluidics Station 450 (Cat#00-0079, Affymetrix) followed the manufacturer' s instructions. Slides were scanned by GeneChip Scanner 3000 (Cat#00-00212, Affymetrix) and Command Console Software 3.1 (Affymetrix) with default settings. Raw data were normalized by MAS 5.0 algorithm, GeneSpring Software 11.0 (Agilent technologies).The expression data were deposited in Gene Expression Omnibus (GEO accession number GSE65210).

### Gene set enrichment analysis

GSEA was performed using the GSEA program provided by the Broad Institute (http://www.broadinstitute.org/gsea/index.jsp). GSEA was used for comparing the expression of a set of E2F1 target genes (gene sets named E2F1_UP. V1 UP from Molecular Signature Database) between POH1-High (expression>average) and POH1-Low (expression<average) tumours, and assesses the relative enrichment of E2F1 positive regulated genes in these two groups.

### Statistical analysis

The differences in the results between groups were compared using *t*-test and ANOVA test. Analysis in tissue samples was performed using the PASW 18.0 Statistical program (SPSS). Difference between the protein levels within tumour and non-tumoral tissues were analysed using the Wilcoxon signed-rank test. The correlation between protein expression levels was analysed using the Spearman correlation test. Associations between protein expression and tumour stages and grades were assessed by the Kruskal–Wallis test and Holm–Sidak's multiple comparisons test. All *P* values <0.05 were considered significant.

## Additional information

**Accession codes:** The microarray data have been deposited in the Gene Expression Omnibus under accession code GSE65210.

**How to cite this article:** Wang, B. *et al.* POH1 deubiquitylates and stabilizes E2F1 to promote tumour formation. *Nat. Commun.* 6:8704 doi: 10.1038/ncomms9704 (2015).

## Supplementary Material

Supplementary Information Supplementary Figures 1-17, Supplementary Table 1 and Supplementary References
