# Supplementary material for: POH1 deubiquitylates and stabilizes E2F1 to promote tumour formation
Source: Nat Commun. 2015 Oct 29;6:8704. doi: 10.1038/ncomms9704 (PMC4846323; doi:10.1038/ncomms9704)
Supplement: Supplementary Information — Supplementary Figures 1-17, Supplementary Table 1 and Supplementary References [file ncomms9704-s1.pdf]

Supplementary Figures

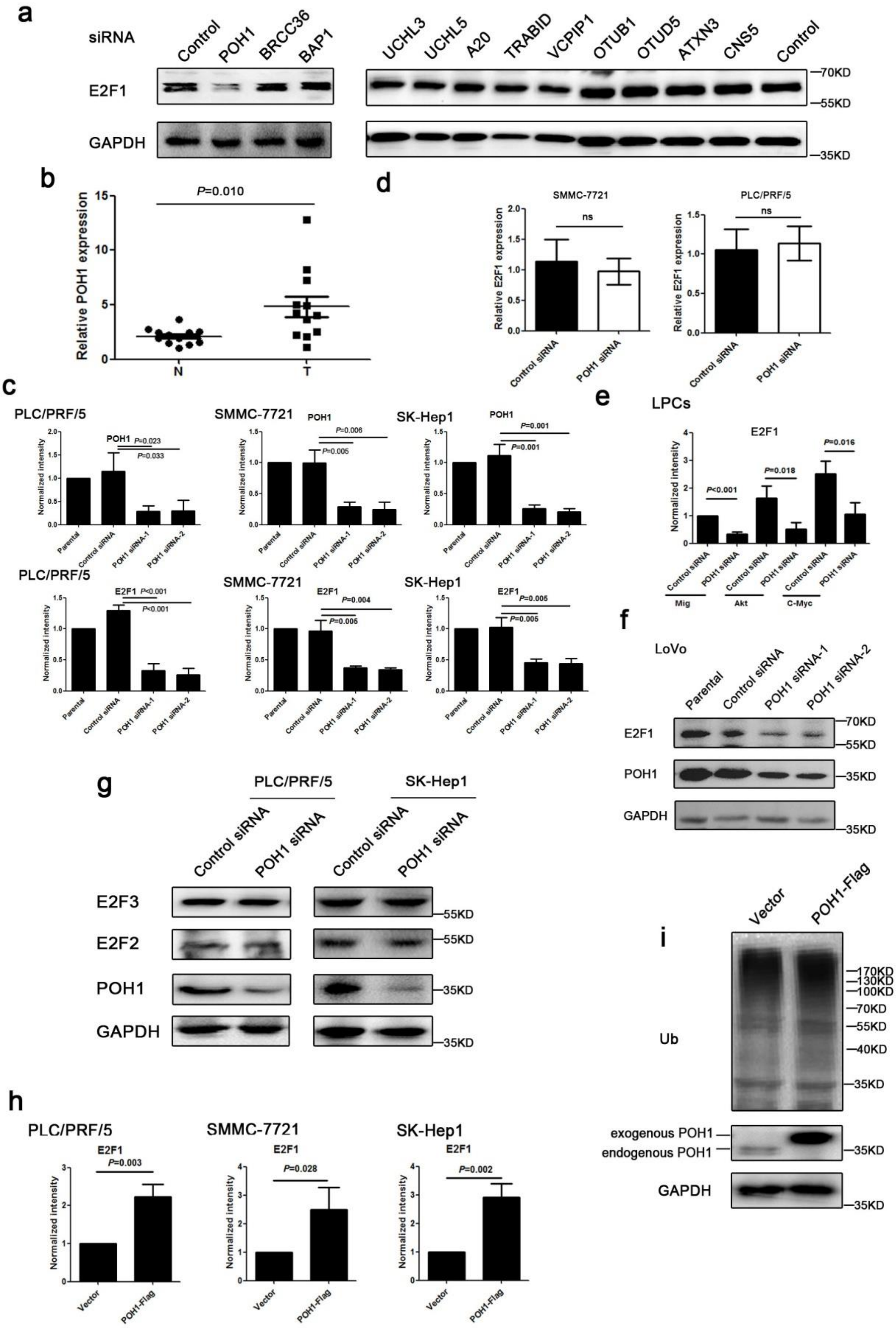

Supplementary Fig 1

Supplementary Figure 1. Screening the DUB that regulates E2F1. (a) The representative images showing E2F1 protein levels in PLC/PRF/5 cells with the

transfection of the DUBs siRNAs. **(b)** Real-time RT-PCR analysis of mRNA levels of POH1 in 12 pairs of HCCs and non-tumoral liver tissues. Data are mean ± s.e.m. (by paired *t*-test analysis, *P* value is shown in the graph) **(c)** Quantitative and statistical analysis of the western blotting assays in Fig. 1b. Experiments were independently performed three times with similar results. In each independent experiment, the intensity of the band was quantified by densitometry analysis and normalized to the loading control. The relative values were then calculated. Data are mean ± s.d. (by *t*-test analysis, *P* values are shown in the graph, n=3). **(d)** Levels of E2F1 mRNA in the SMMC-7721 and PLC/PRF/5 cells transfected with control siRNA or POH1 siRNA were determined by real-time RT-PCR. Data are mean ± s.d. (by *t*-test analysis, no significance, n=3). **(e)** Quantitative and statistical analysis of the western blotting assays in Fig. 1c. Data are mean ± s.d. (by *t*-test analysis, *P* values are shown in the graph, n=3). **(f)** Immunoblotting of E2F1 in control siRNA or POH1 siRNAs transfected colon cancer cell line LoVo. **(g)** Immunoblotting of E2F2, E2F3 and POH1 in PLC/PRF/5 and SK-Hep1 cells transfected with Control siRNA and POH1 siRNA. **(h)** Quantitative and statistical analysis of the western blotting assays in Fig. 1d. Data are mean ± s.d. (by *t*-test analysis, *P* values are shown in the graph, n=3). **(i)** Western blotting analysis of the total ubiquitination-modified proteins in SMMC-7721 cells with or without POH1-Flag expression.

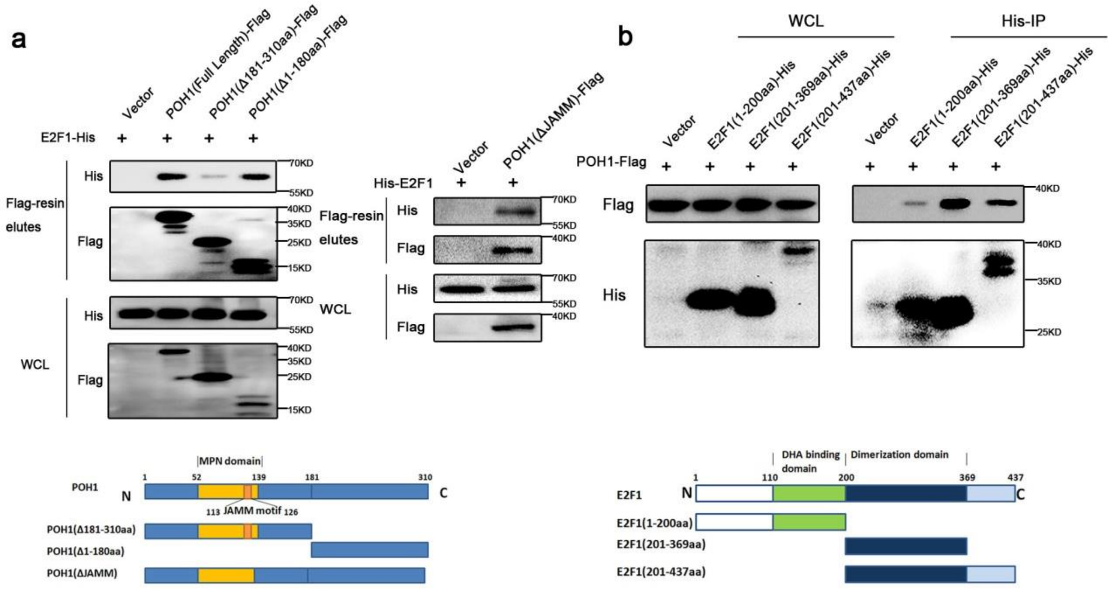

Supplementary Fig 2

Supplementary Figure 2. Analyzing the domains of POH1 and E2F1 required for their interaction. (a) HEK293T cells were co-transfected with the full-length POH1-Flag, or the indicated truncated POH1-Flag along with the E2F1-His.The different forms of POH1-Flag proteins were mmunoprecipited using Flag M2 beads, and the co-immunoprecipited E2F1-His proteins were detected by western blot. **(b)** HEK293T cells were co-transfected with the POH1-Flag along with the indicated truncated mutants of E2F1-His. The different truncated mutants of E2F1-His were immunoprecipited by anti-His antibody, and the co-immunoprecipited POH1-Flag proteins were detected using western blot.

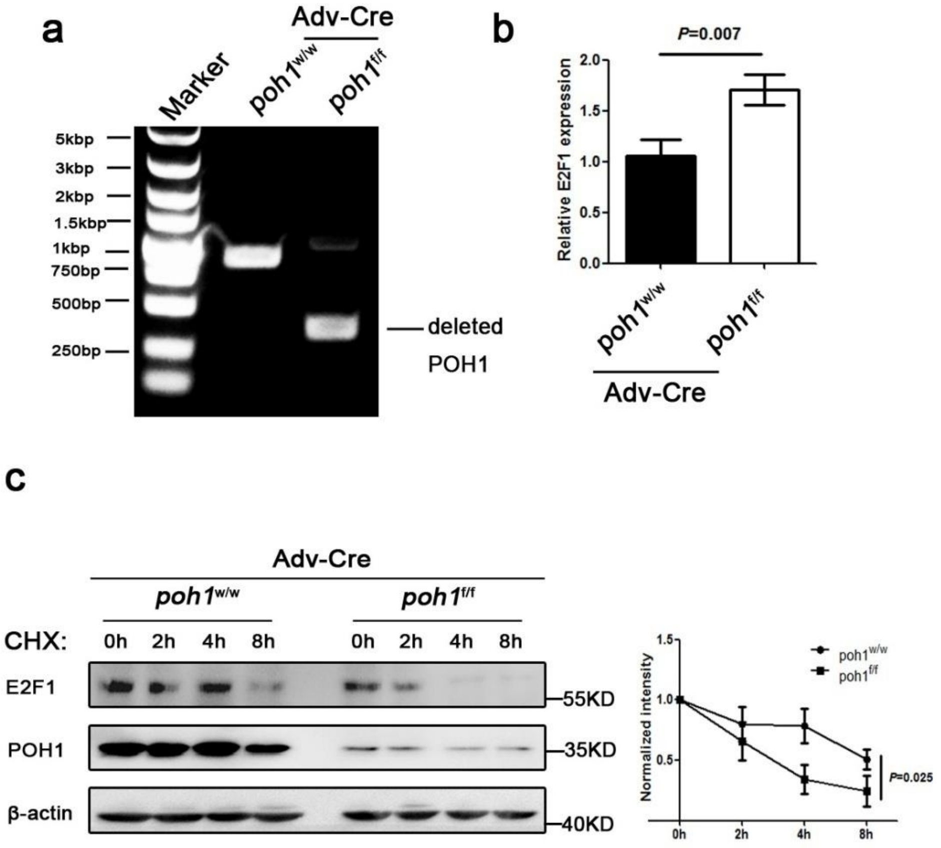

Supplementary Fig 3

Supplementary Figure 3. POH1 deletion in mouse embryonic fibroblasts destabilized E2F1 protein. (a) PCR analysis of genomic DNA demonstrating that the floxed *poh1* alleles are efficiently deleted in *poh1*$^{f/f}$ MEFs by two rounds of Adv-Cre (1×10^{8} PFU) infection. **(b)** Real-time PCR analysis of mRNA levels of E2F1 in *poh1*$^{w/w}$ and *poh1*$^{f/f}$ MEFs upon Adv-Cre infection. Data are mean ± s.d. (by *t*-test analysis, *P* value is shown in the graph, n=3). **(c)** After Adv-Cre infection, the *poh1*$^{w/w}$ and *poh1*$^{f/f}$ MEFs were treated with CHX (100μg ml^{-1}) for the indicated time points. The cell lysates were examined by immunoblotting using the indicated antibodies (left panel). A plot of normalized amount of E2F1 protein was shown (right panel). Data are mean ± s.d. (by ANOVA analysis, *P* value is shown in the graph, n=3).

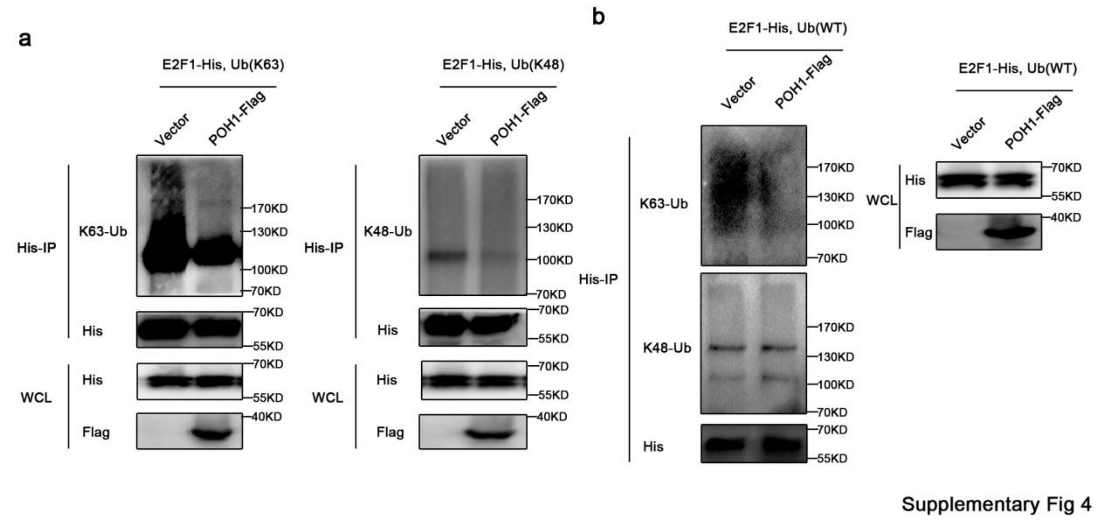

Supplementary Figure 4. POH1 regulates K63-linked polyubiquitination of E2F1. (a) HEK293T cells were co-transfected with E2F1-His, vector, or the POH1-Flag, and UB (K63)-HA or UB (K48)-HA constructs. Cell lysates were immunoprecipitated with anti-His antibody and immunoblotted with the K63-chains or K48-chains specific antibodies. **(b)** HEK293T cells were co-transfected with E2F1-His, vector, or the POH1-Flag, and UB (WT)-HA. Cell lysates were immunoprecipitated with anti-His antibody and immunoblotted with the K63-chains or K48-chains specific antibodies.

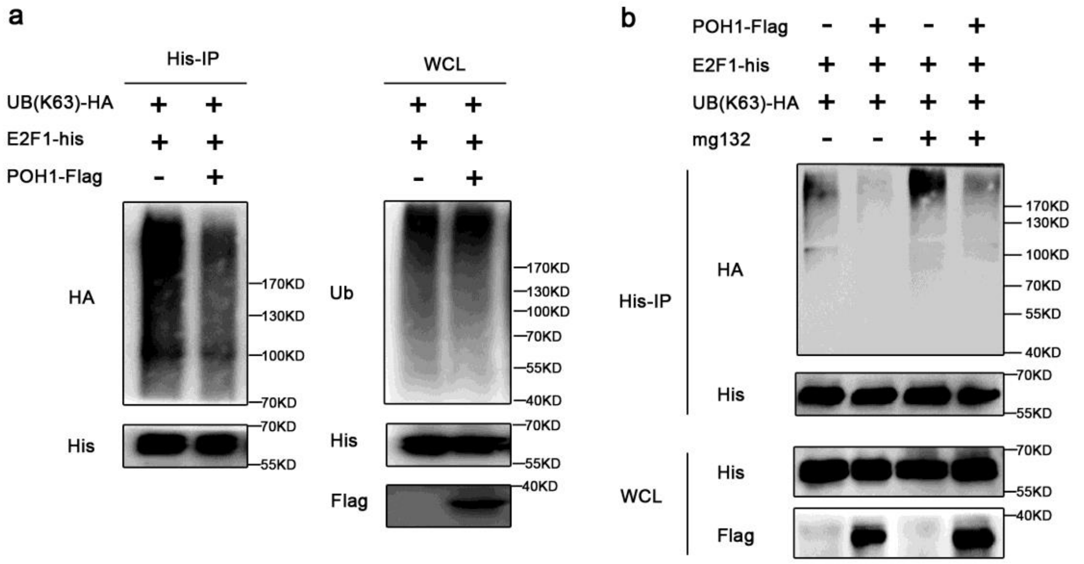

Supplementary Fig 5

Supplementary Figure 5. POH1 overexpression results in E2F1 deubiquitination but does not change total levels of polyubiquitinated proteins. (a) HEK293T cells were co-transfected with E2F1-His and UB (K63)-HA along with or without POH1-Flag. Cell lysates were immunoprecipitated with anti-His antibody and immunoblotted with the indicated antibodies. The whole cell lysates were also immunoblotted to test the total polyubiquitinated proteins. **(b)** HEK293T cells transfected with the indicated plasmids were treated with or without MG132 (10µM) for 6 h. Cell lysates were immunoprecipitated using anti-His antibody. The levels of polyubiquitinated proteins were detected using anti-HA antibody.

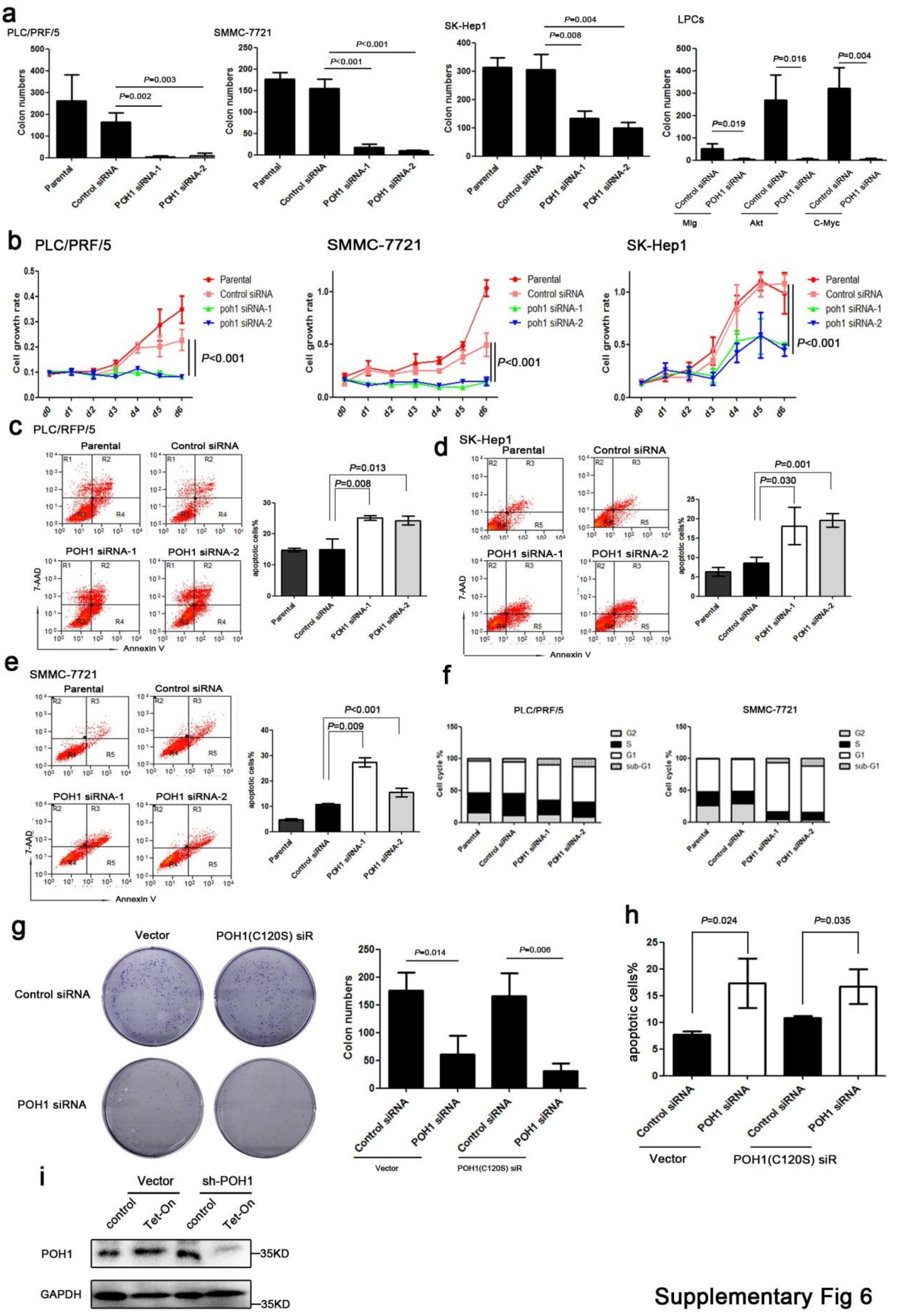

Supplementary Figure 6. Knockdown of POH1 promotes HCC cell apoptosis and cell cycle arrest. (a) Quantitative and statistical analyses of the colony formation data in Fig. 4a-b. Data are mean ± s.d. (by *t*-test analysis, *P* values are shown in the graph, n=3).

(b) PLC/PRF/5, SMMC-7721 and SK-Hep1 cells with or without transfection of control siRNA or POH1siRNAs were examined by MTT assay. Data are mean ± s.d. (by ANOVA analysis, *P* values are shown in the graph, n=3). **(c. d. e)** PLC/PRF/5 **(c)**, SMMC-7721 **(d)** and SK-Hep1 **(e)** cells were untransfected or transfected with control siRNA, POH1 siRNA-1 or POH1 siRNA-2, respectively. Cells were harvested 96h post-transfection and subjected to apoptosis analysis. Data are mean ± s.d. (by *t*-test analysis, *P* values are shown in the graph, n=3). **(f)** PLC/PRF/5 and SMMC-7721 cells were untransfected or transfected with control siRNA, POH1 siRNA-1 or POH1 siRNA-2 for 72h and then subjected to cell cycle analysis. The representative images are shown. **(g.h)** SMMC-7721 cells with or without RNAi-resistant POH1(C120S)-Flag were transfected with control siRNA or POH1 siRNA for 72 h, then cells were seeded in 6-well plates (2000 cells per well) for colony formation assay for another 10 days. The representative image is shown on the left panel. Quantitative and statistical analysis of the colony formation data is shown on the right panel. Data are mean ± s.d. (by *t*-test analysis, *P* values are shown in the graph, n=3). **(g)** or examined by apoptosis assay. Data are mean ± s.d. (by *t*-test analysis, *P* values are shown in the graph, n=3) **(h)**. **(i)** Western bolt assay confirmed the efficiency of doxycyclin-induced knockdown of POH1 in the sh-POH1 expressing SK-Hep1 cells.

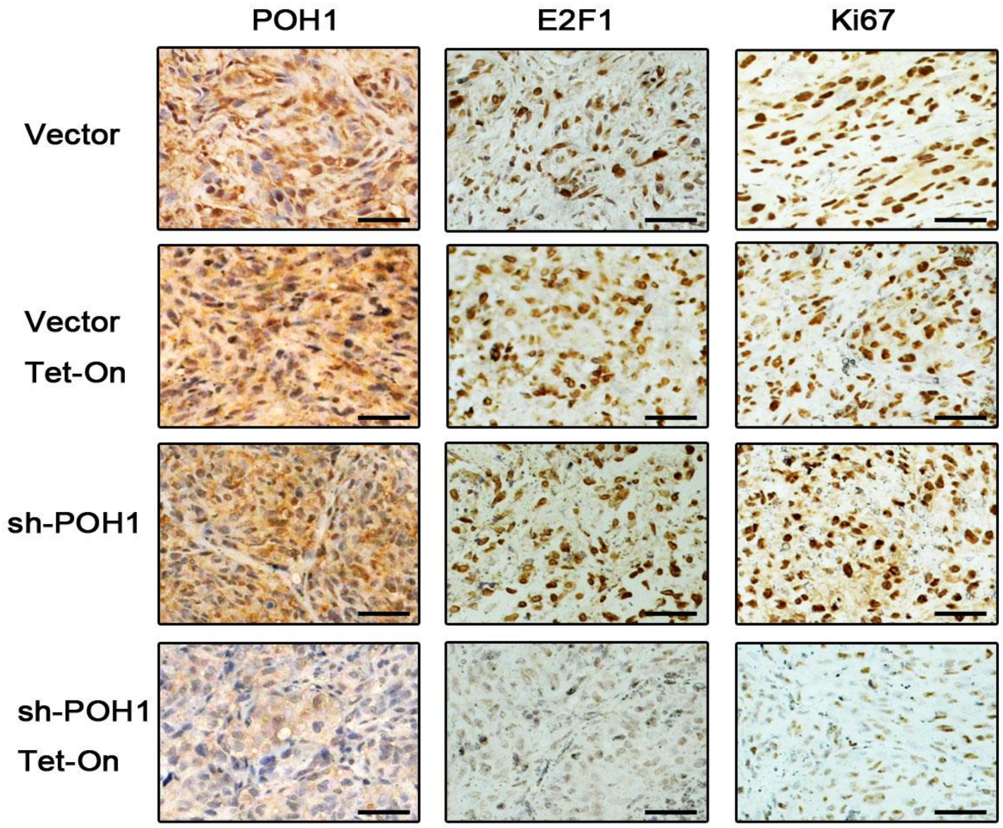

Supplementary Fig 7

Supplementary Figure 7. Knockdown of POH1 results in reduced E2F1 and Ki67 expression in xenograft tumor cells. Immunohistochemical staining of POH1 and E2F1 in xenograft tumor tissues. The degree of tumor proliferation was determined by Ki67 staining, Bar=100μm.

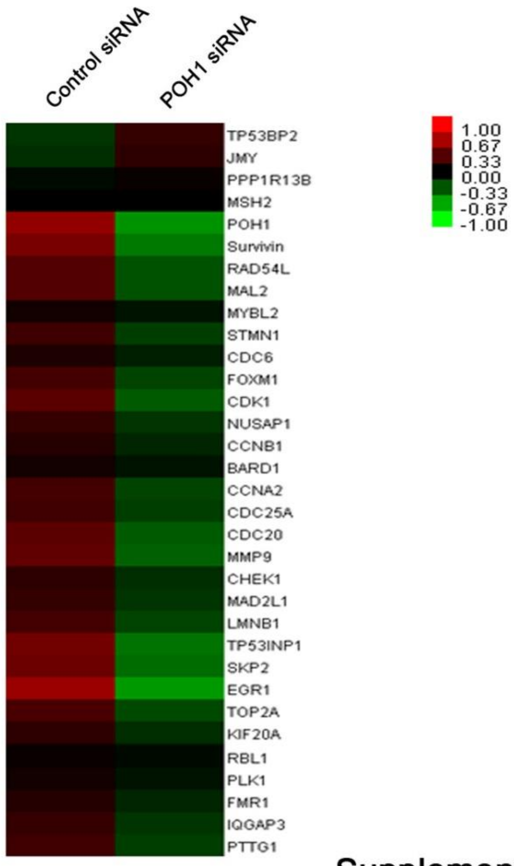

Supplementary Fig 8

Supplementary Figure 8. POH1 regulates a set of E2F1 target genes. The total RNA extracted from the control or POH1 knockdown SMMC-7721 cells were subjected to whole-genome transcripts microarray analysis (Human U133 Plus 2.0 microarray).The heatmap depicts a compilation of several E2F1 targets.

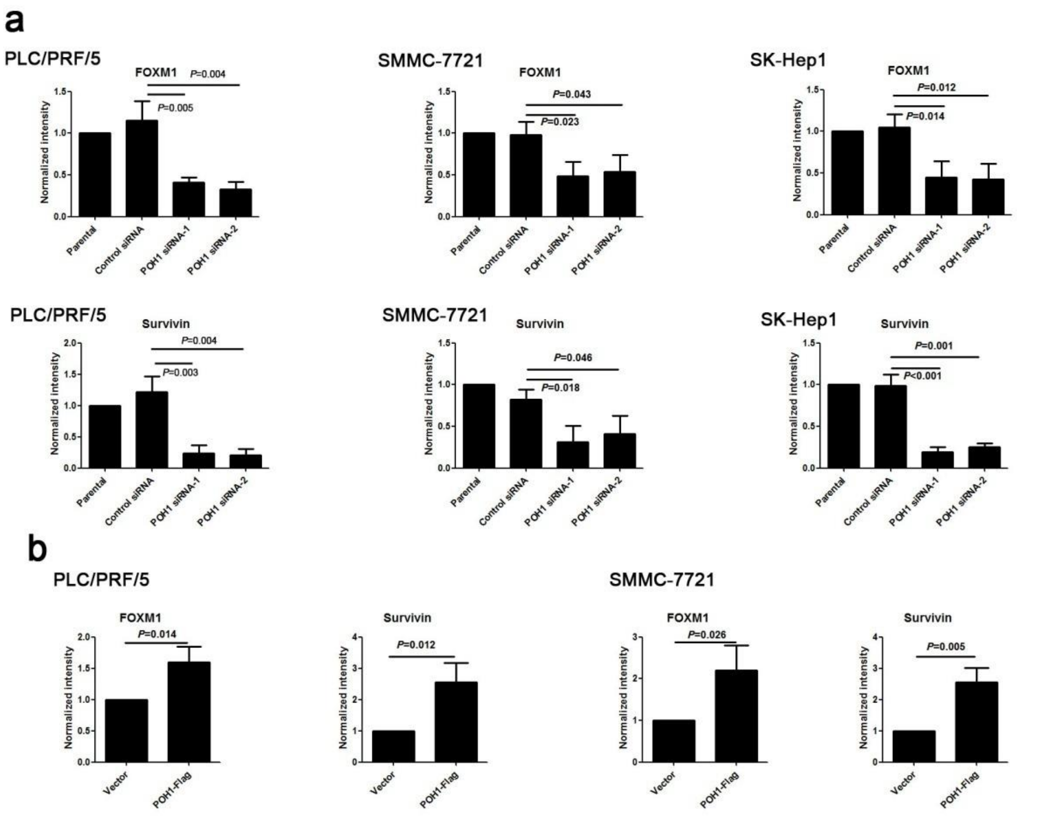

Supplementary Fig 9

Supplementary Figure 9. Quantitative and statistical analyses of the indicated immunoblots (a. b) Quantitative and statistical analyses of the western blotting assays in Fig. 5b **(a)** and Fig. 5c **(b)**. Data are mean ± s.d. (by *t*-test analysis, *P* values are shown in the graph, n=3).

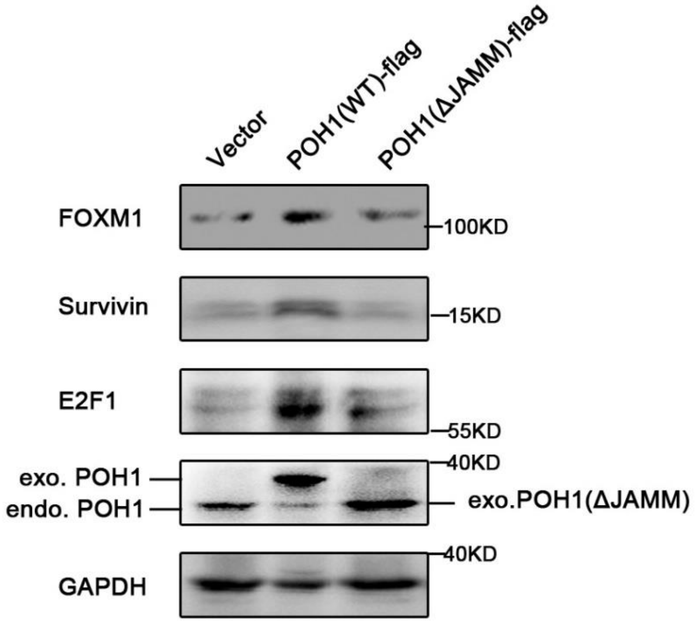

Supplementary Fig 10

Supplementary Figure 10. Overexpression of wild-type POH1 but not the enzyme-dead mutant enhances the expression of E2F1, Survivin and FOXM1 proteins. PLC/PRF/5 cells overexpressing POH1(WT)-Flag and POH1(ΔJAMM)-Flag cells were subjected to western blot assay to determine the E2F1, Survivin and FOXM1 protein levels.

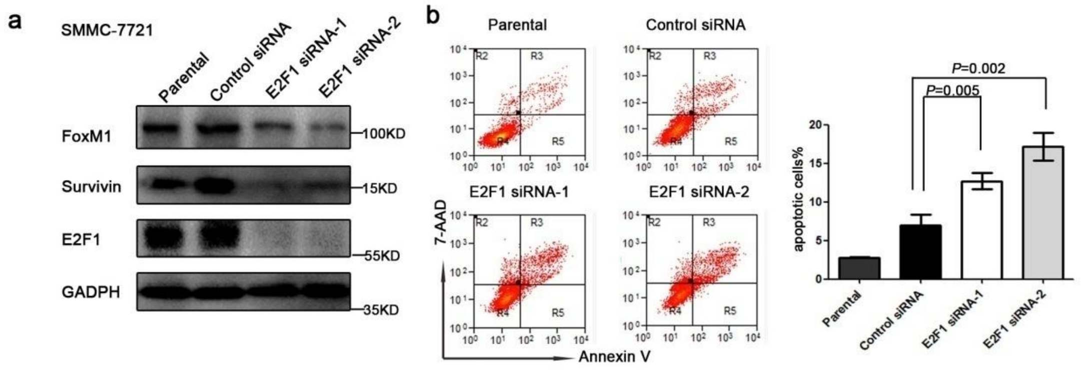

Supplementary Fig 11

Supplementary Figure 11. Knockdown of E2F1 induces cell apoptosis and decreases the levels of Survivin and FOXM1 proteins. (a) The SMMC-7721 cells were untransfected or transfected with control siRNA, E2F1 siRNA-1 or E2F1 siRNA-2. The cells were subjected to western blot assay to determine Survivin and FOXM1 protein levels. **(b)** Apoptosis analysis of SMMC-7721 cells untransfected or transfected with control siRNA, E2F1 siRNAs. Data are mean ± s.d. (by *t*-test analysis, *P* values are shown in the graph, n=3).

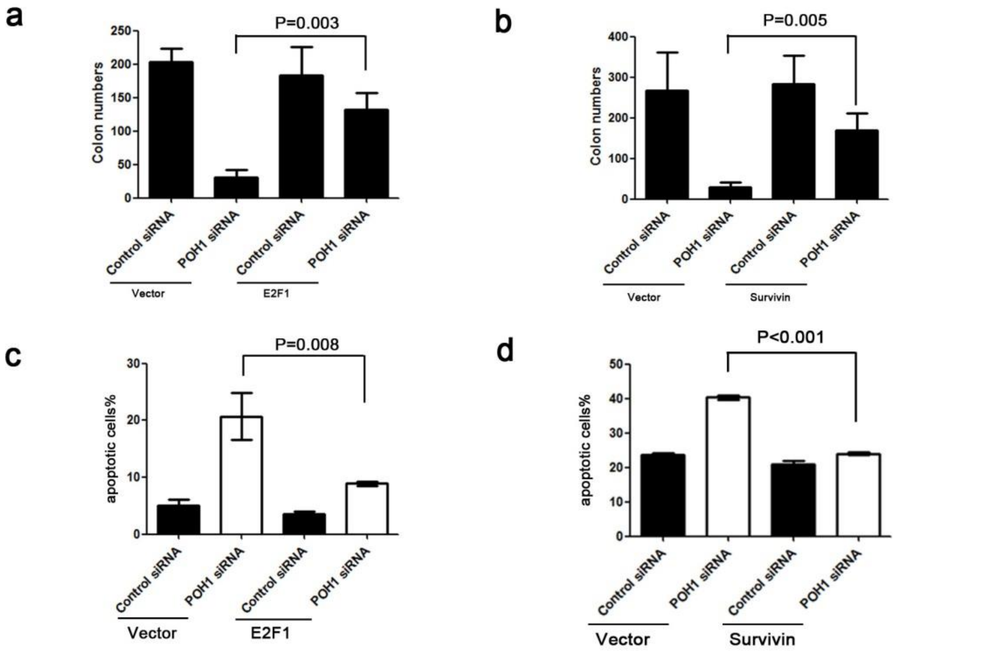

Supplementary Fig 12

Supplementary Figure 12. Restoration of E2F1 and Survivin rescues POH1 depletion-induced growth arrest and apoptosis. (a. b) Quantitative and statistical analyses of the colony formation data in Fig. 5f **(a)** and Fig. 5h **(b)**. Data are mean ± s.d. (by *t*-test analysis, *P* values are shown in the graph, n=3). **(c. d)** PLC/PRF/5 cells overexpressing E2F1**(c)** or Survivin**(d)** were transfected with control siRNA or POH1 siRNA for 96h, then the cells were harvested for apoptosis analysis. Data are mean ± s.d. (by *t*-test analysis, *P* values are shown in the graph, n=3).

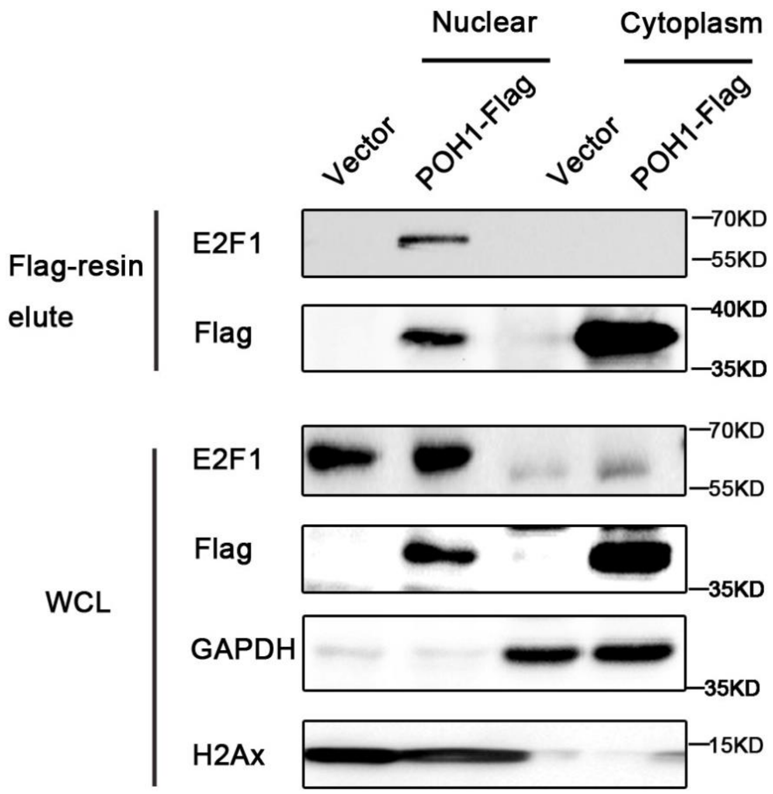

Supplementary Fig 13

Supplementary Figure 13. Nuclear POH1 interacts with E2F1. The nuclear and cytoplasmic fractions of SMMC-7721 cells expressing POH1-Flag were extracted, POH1-Flag proteins were immunoprecipitated using anti-Flag antibody from both fractions and the co-immunoprecipitated E2F1 proteins were tested by western blotting.

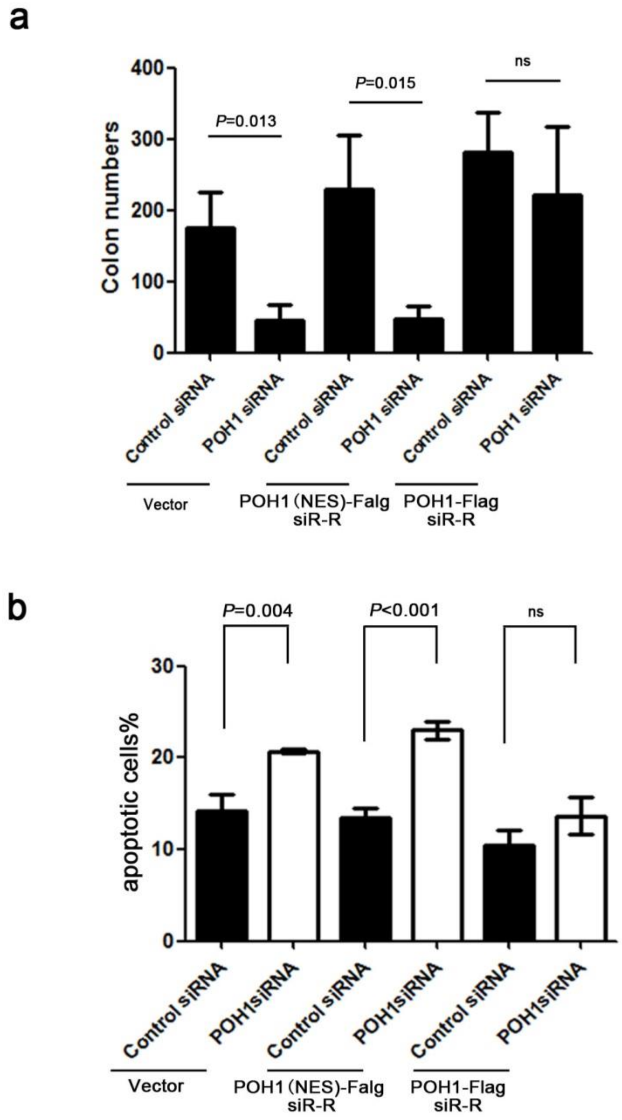

Supplementary Fig 14

Supplementary Figure 14. Cytoplasmic POH1 fails to rescue POH1 knockdown -induced growth arrest and apoptosis. (a) Quantitative and statistical analyses of the colony formation data in Fig. 6h. Data are mean ± s.d. (by *t*-test analysis, *P* values are shown in the graph, n=3). **(b)** SMMC-7721 cells expressing two different forms of POH1 were transfected with control or POH1 siRNA. Four days after transfection, the cells were subjected to apoptosis analysis. Data are mean ± s.d. (by *t*-test analysis, *P* values are shown in the graph, n=3).

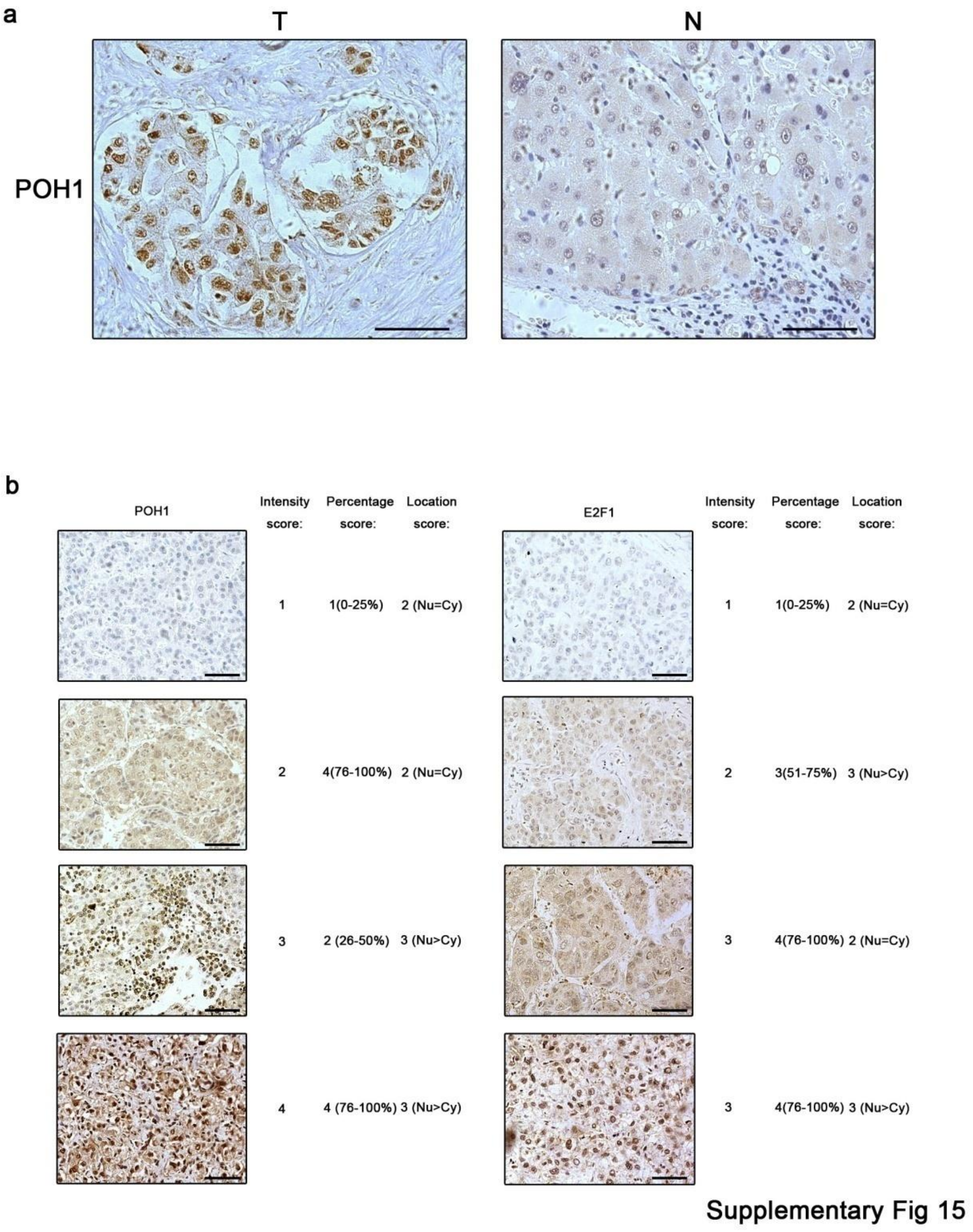

Supplementary Figure 15. Representative images of IHC staining of POH1 and E2F1 in human HCCs. (a) Representative images for POH1 staining in HCC and non-tumoral liver tissues. **(b)** Representative images show the staining of POH1 and E2F1 with distinct intensity, percentage and location scores. Bar=100μm.

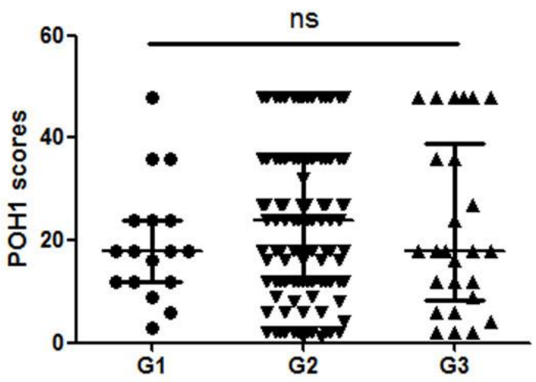

Supplementary Fig 16

Supplementary Figure 16. No correlation between POH1 expression with tumor grades in human HCCs. Correlation between POH1 scores and tumor grades in HCC samples. The scatter dot plot and the median with interquartile range are presented. The *P* value was calculated by Kruskal-Wallis test.

Figure 1.b

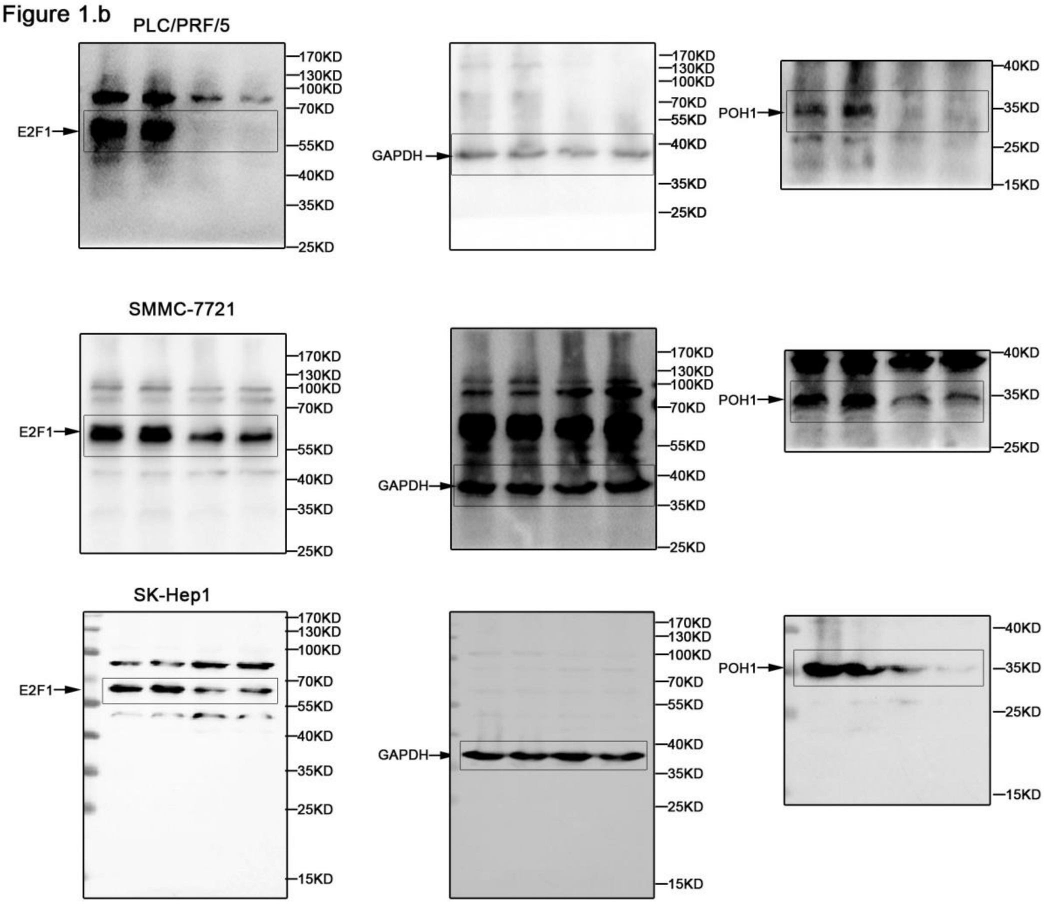

Figure 1.c

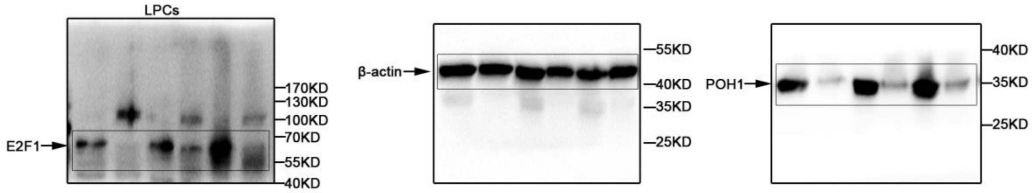

Supplementary Fig. 17

Figure 1.d

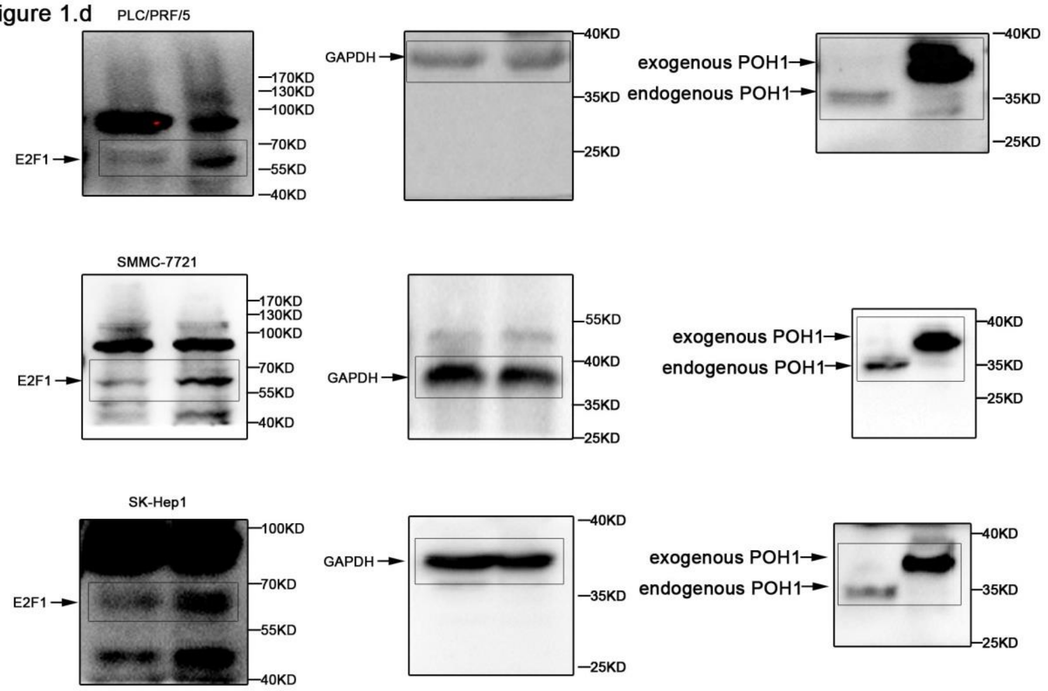

Figure 1.f

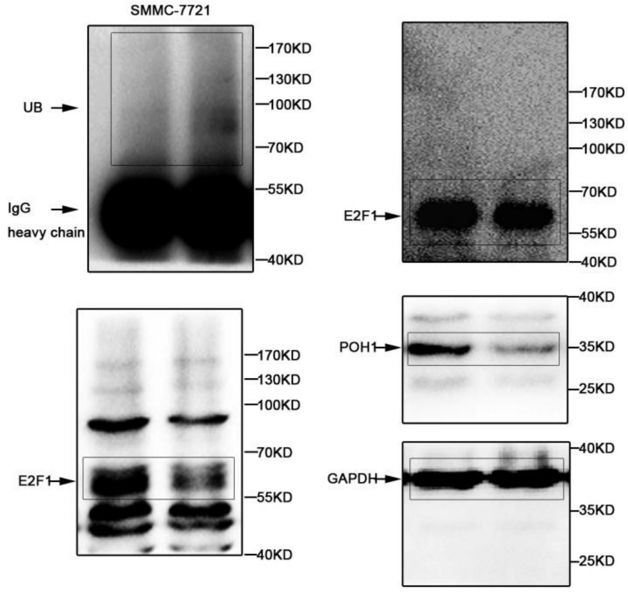

Supplementary Fig. 17 continued

Figure 1.g

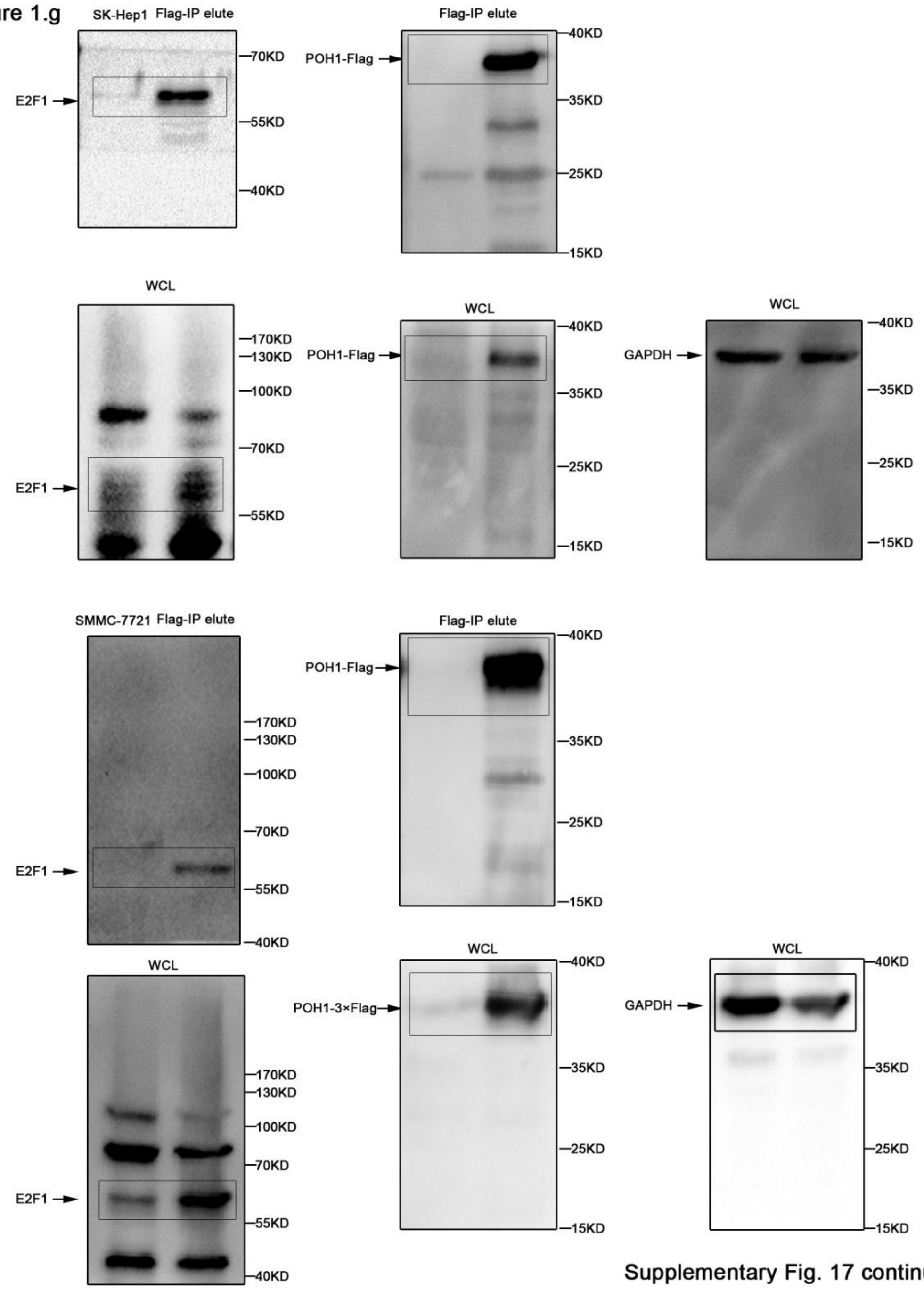

Supplementary Fig. 17 continued

Figure 1.g

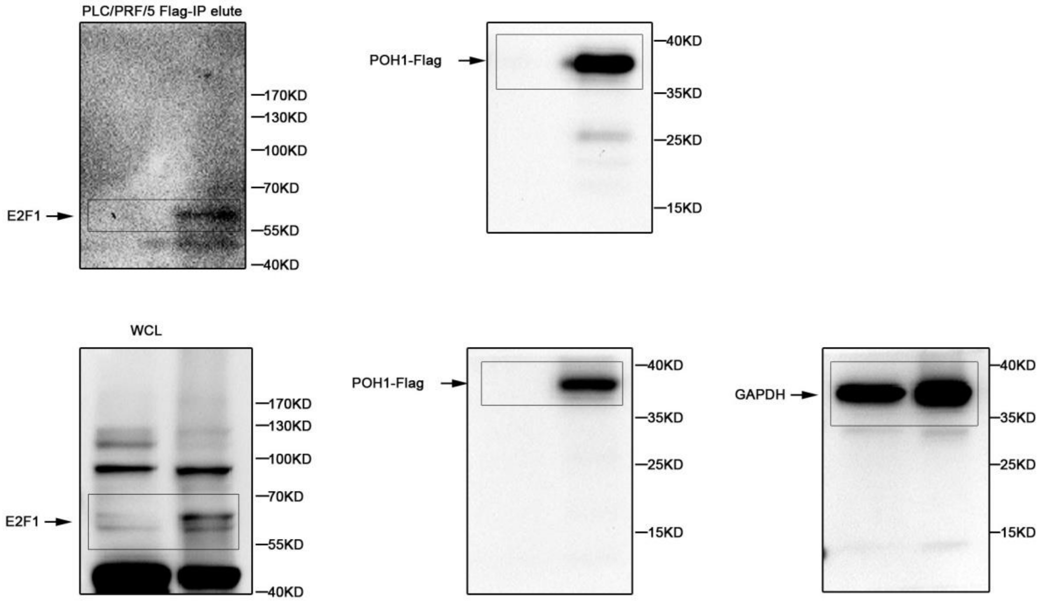

Figure 1.h

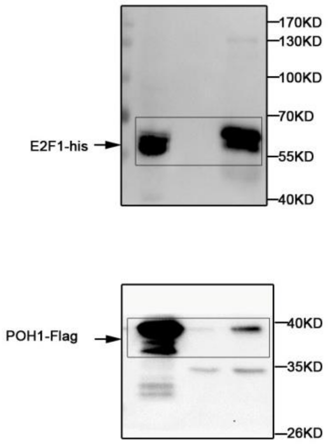

Figure 1.i

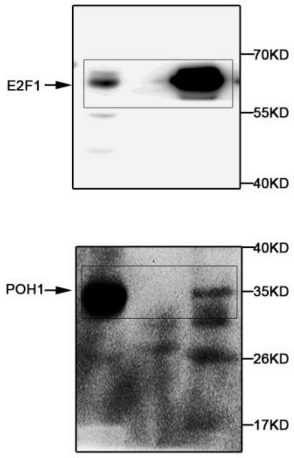

Supplementary Fig. 17 continued

Figure 1.j

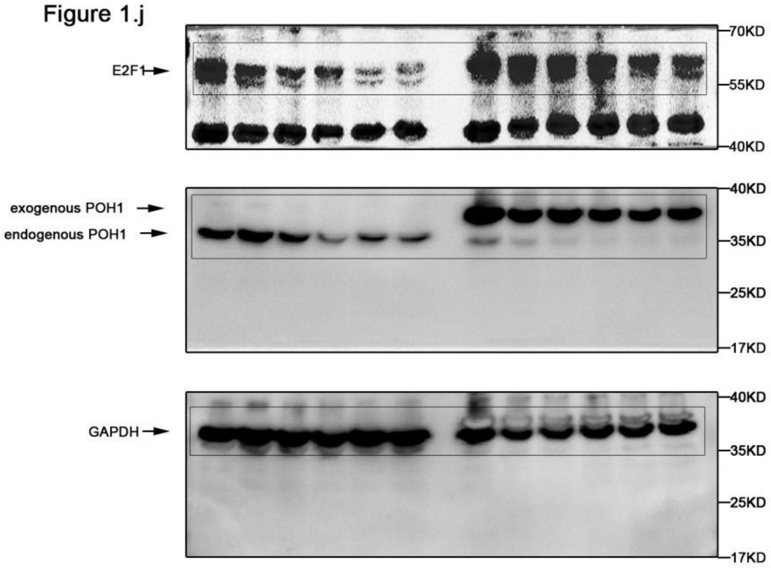

Figure 1.k

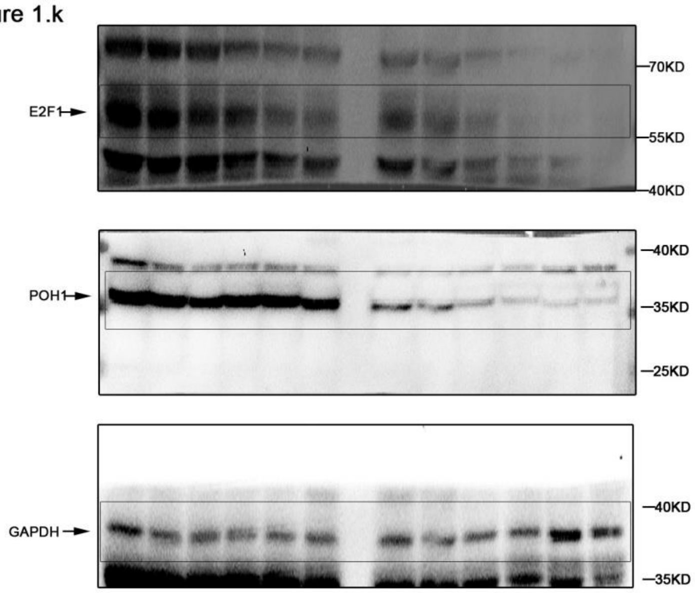

Supplementary Fig. 17 continued

Figure 2.c

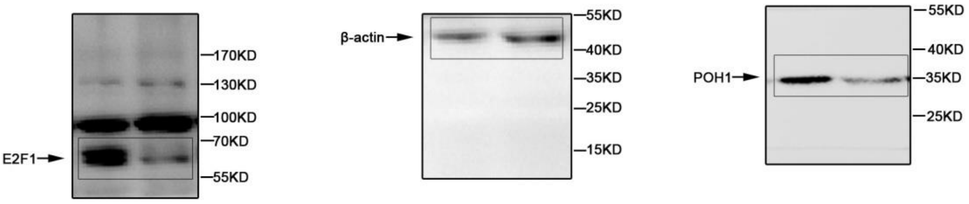

Figure 2.e

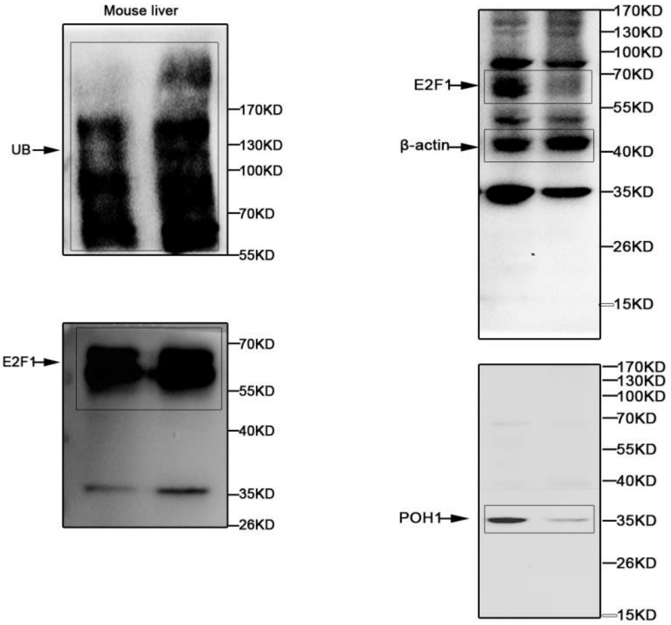

Supplementary Fig. 17 continued

Figure 3.a

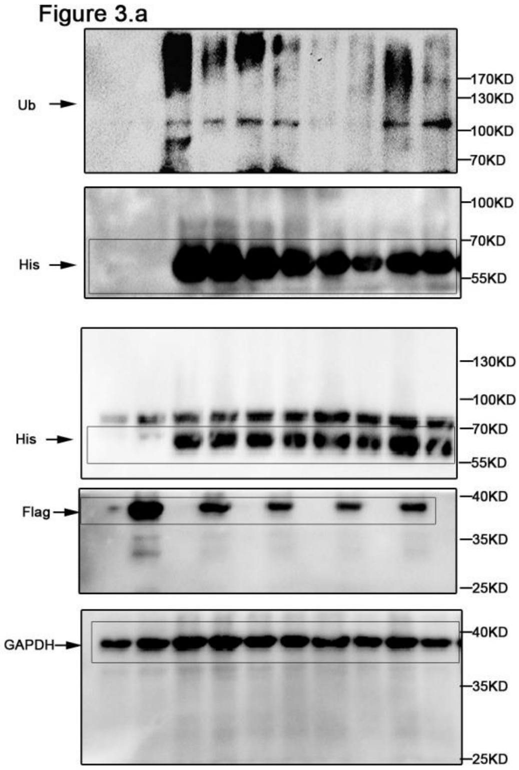

Figure 3.b

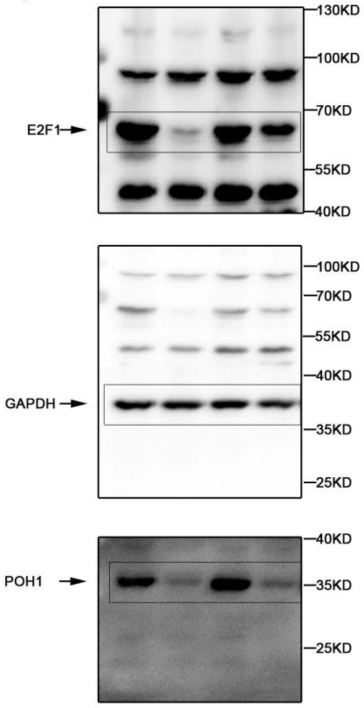

Figure 3.d

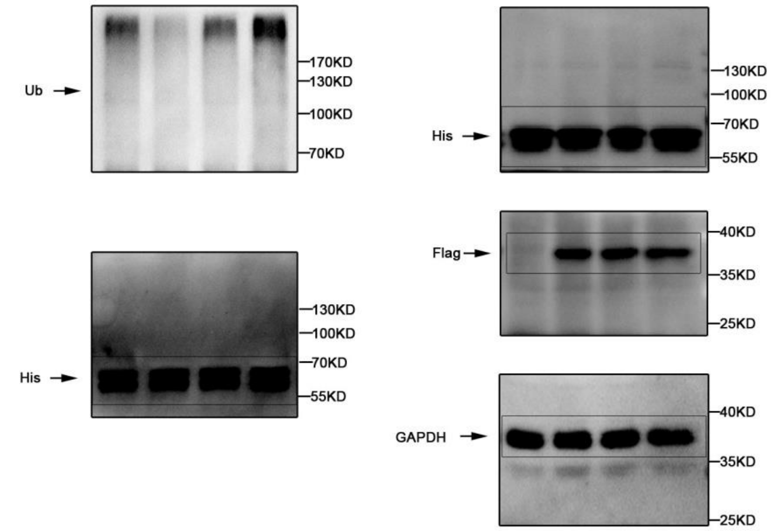

Supplementary Fig. 17 continued

Figure 3.e

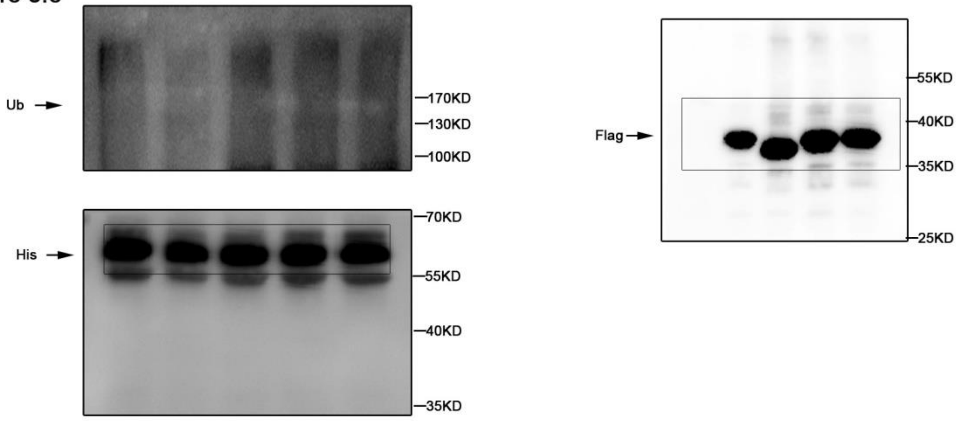

Figure 3.f

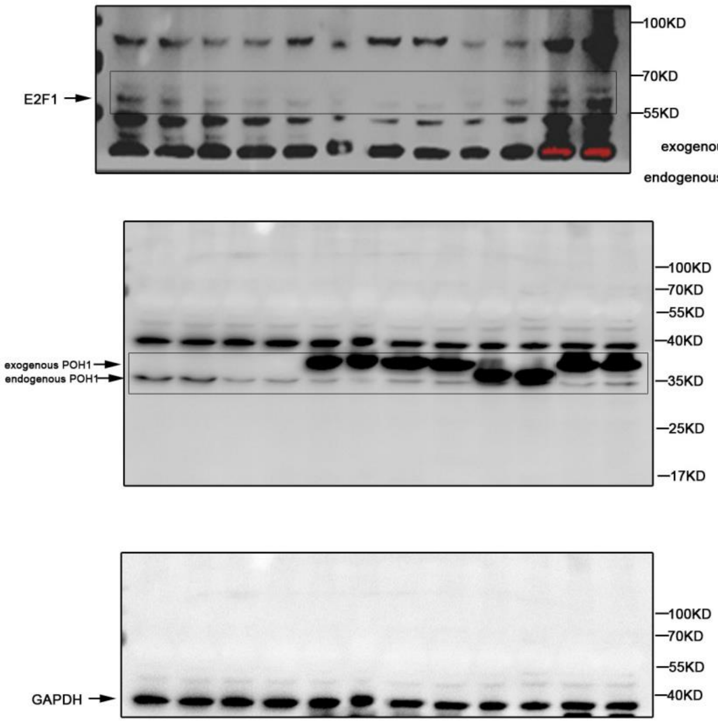

Figure 3.g

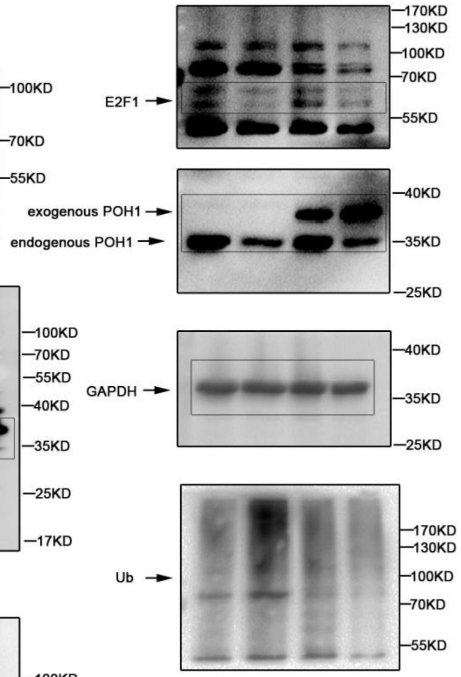

Supplementary Fig. 17 continued

Figure 4. c

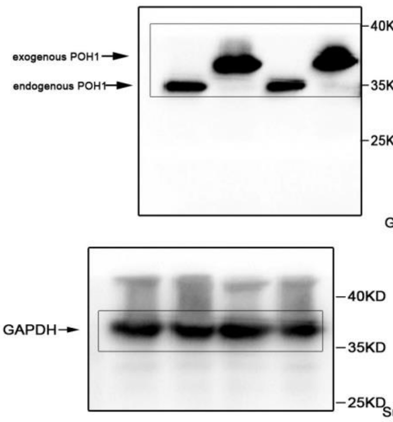

Figure 5. b

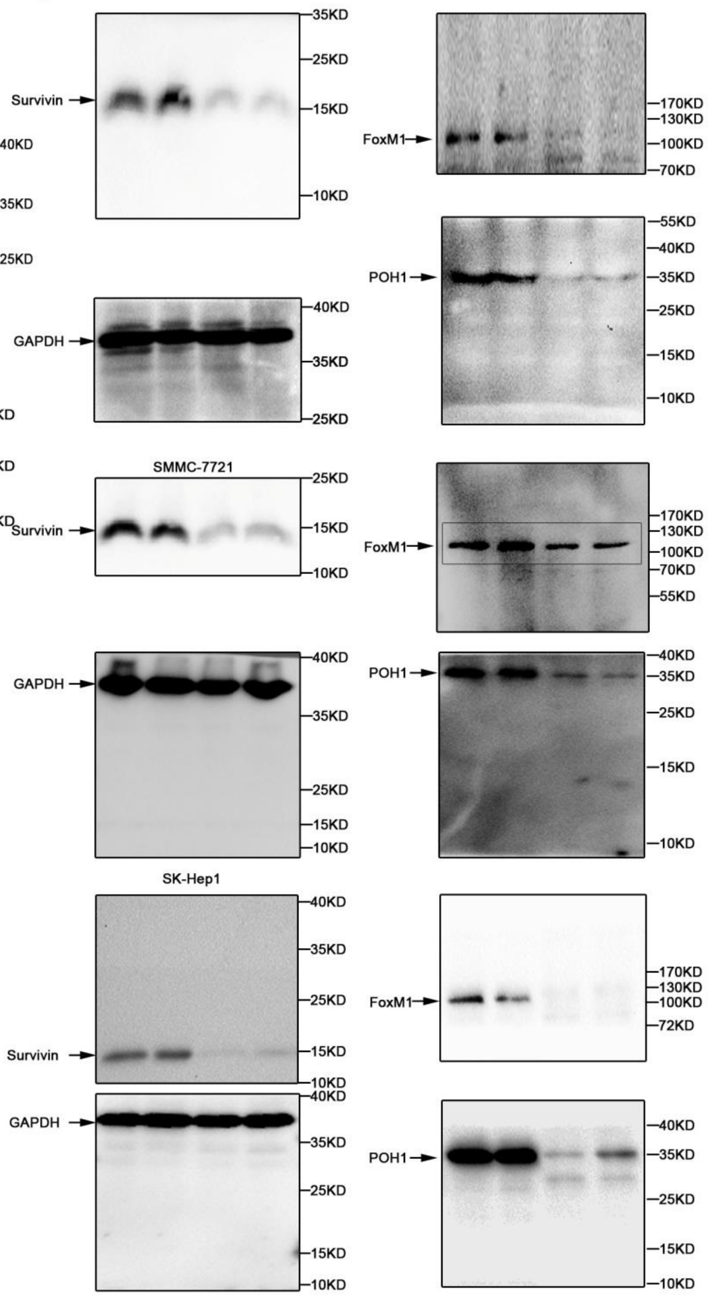

Supplementary Fig. 17 continued

Figure 5.c

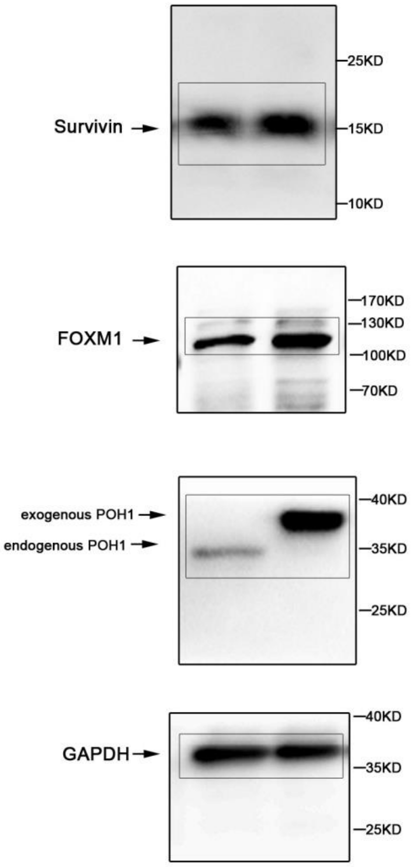

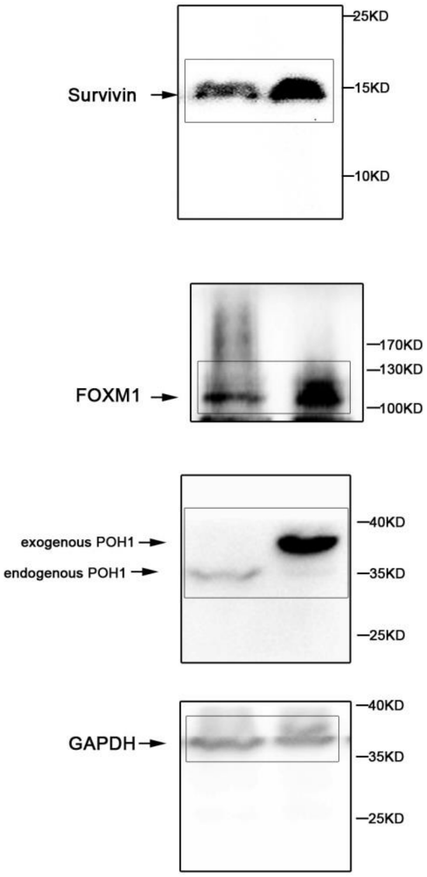

Supplementary Fig. 17 continued

Figure 5.d

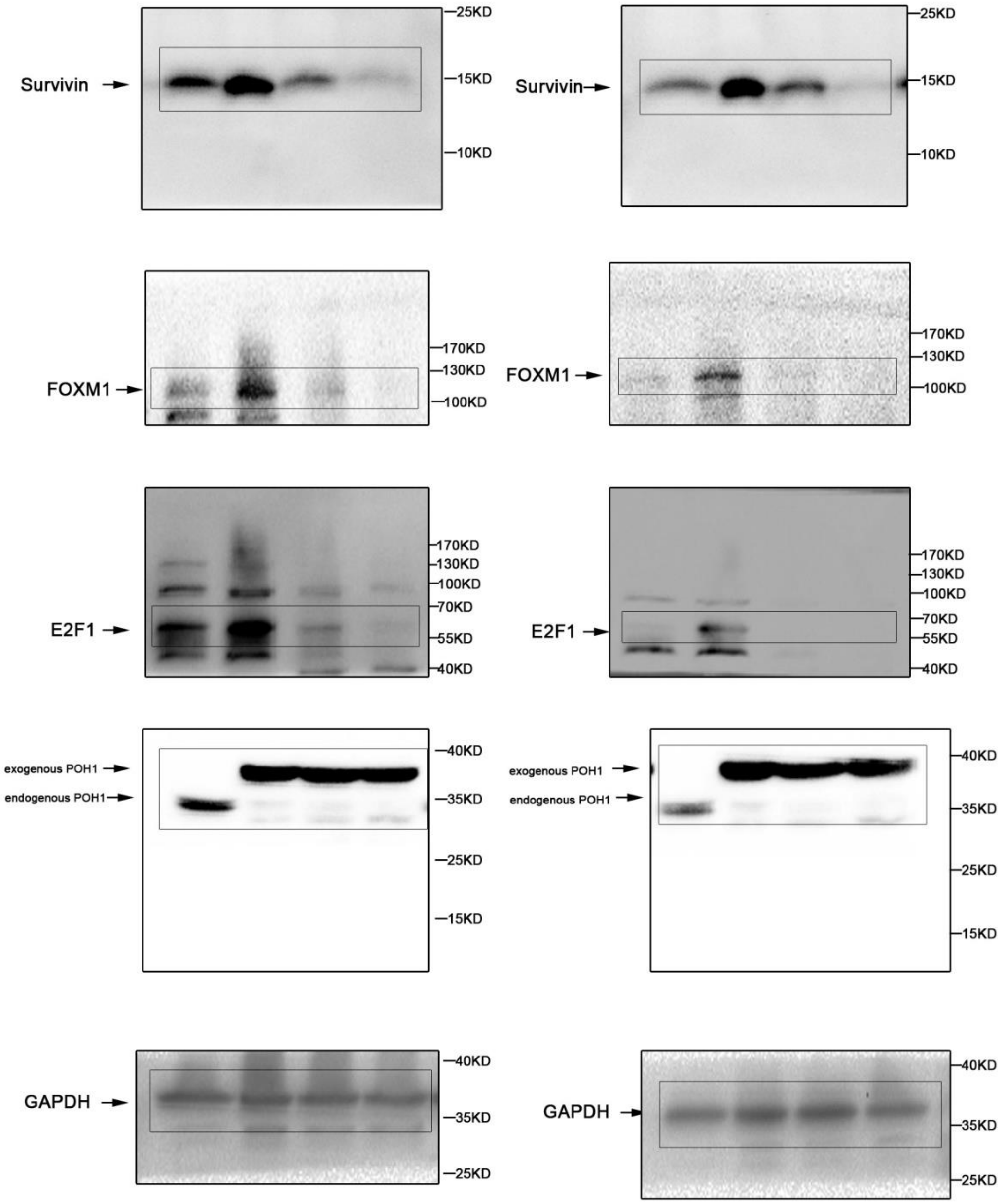

Supplementary Fig.17 continued

Figure 5.e

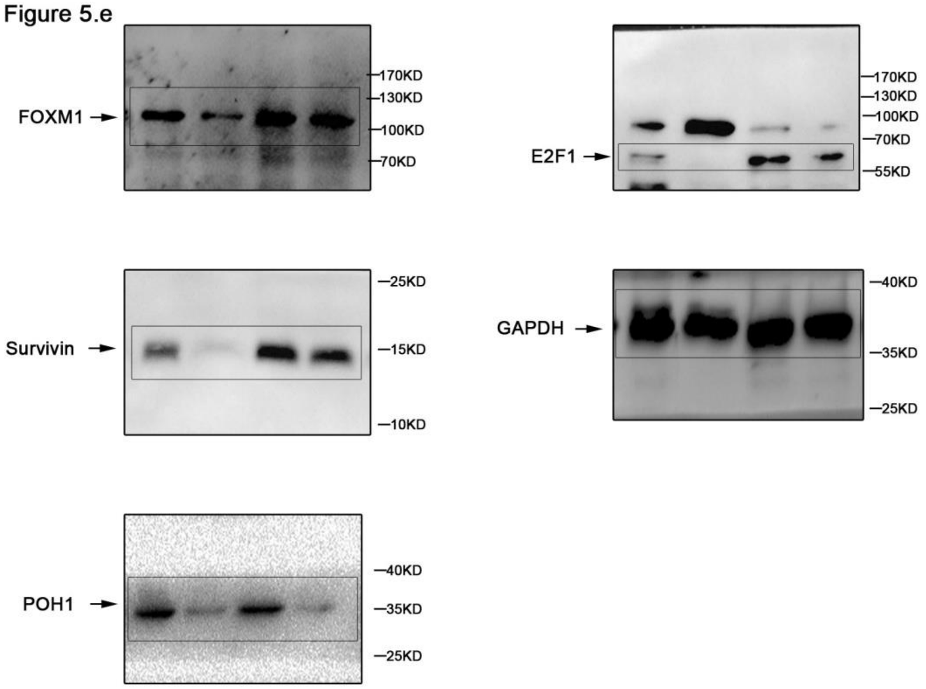

Figure 5.g

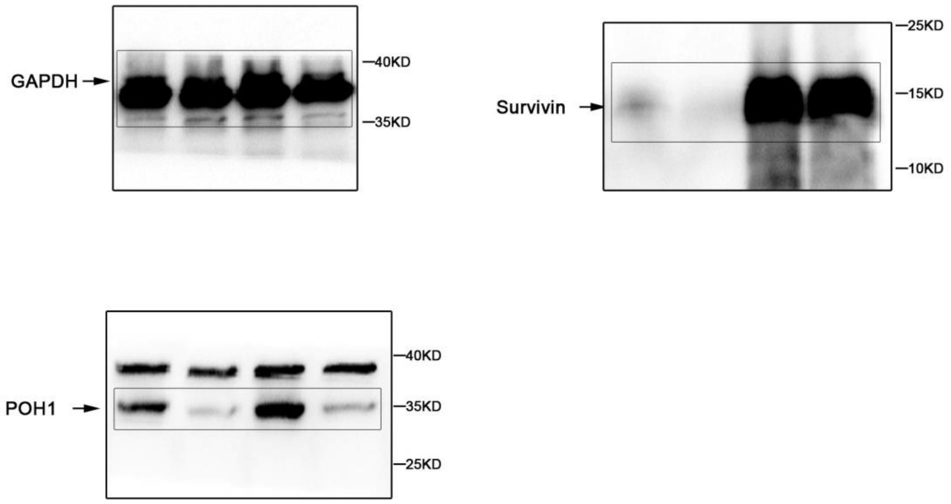

Supplementary Fig.17 continued

Fiugre 6.a

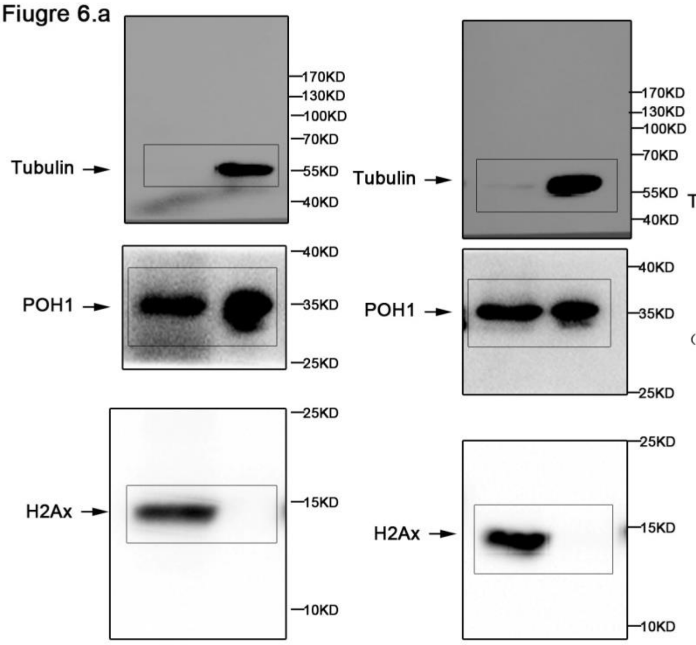

Fiugre 6.b

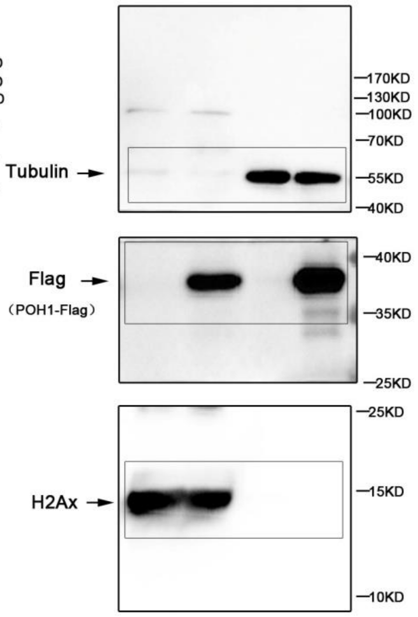

Fiugre 6.e

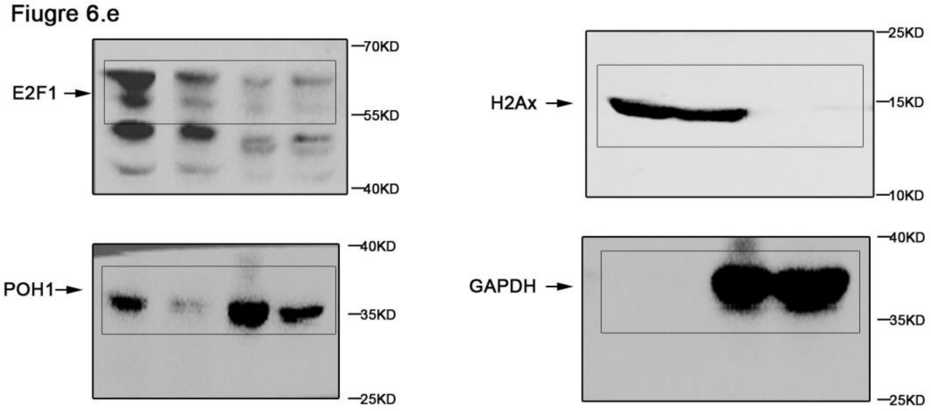

Fiugre 6.f

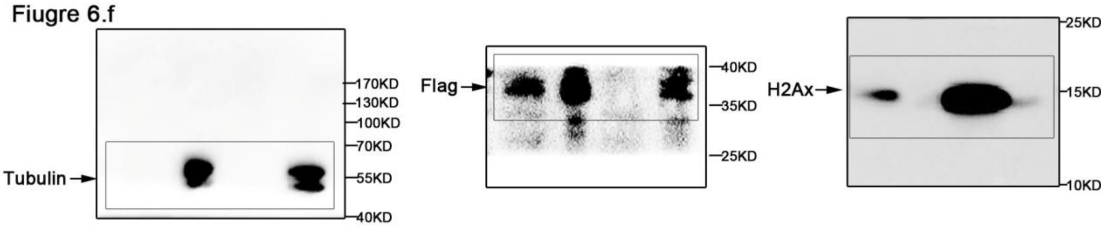

Supplementary Fig.17 continued

Figure 6.g

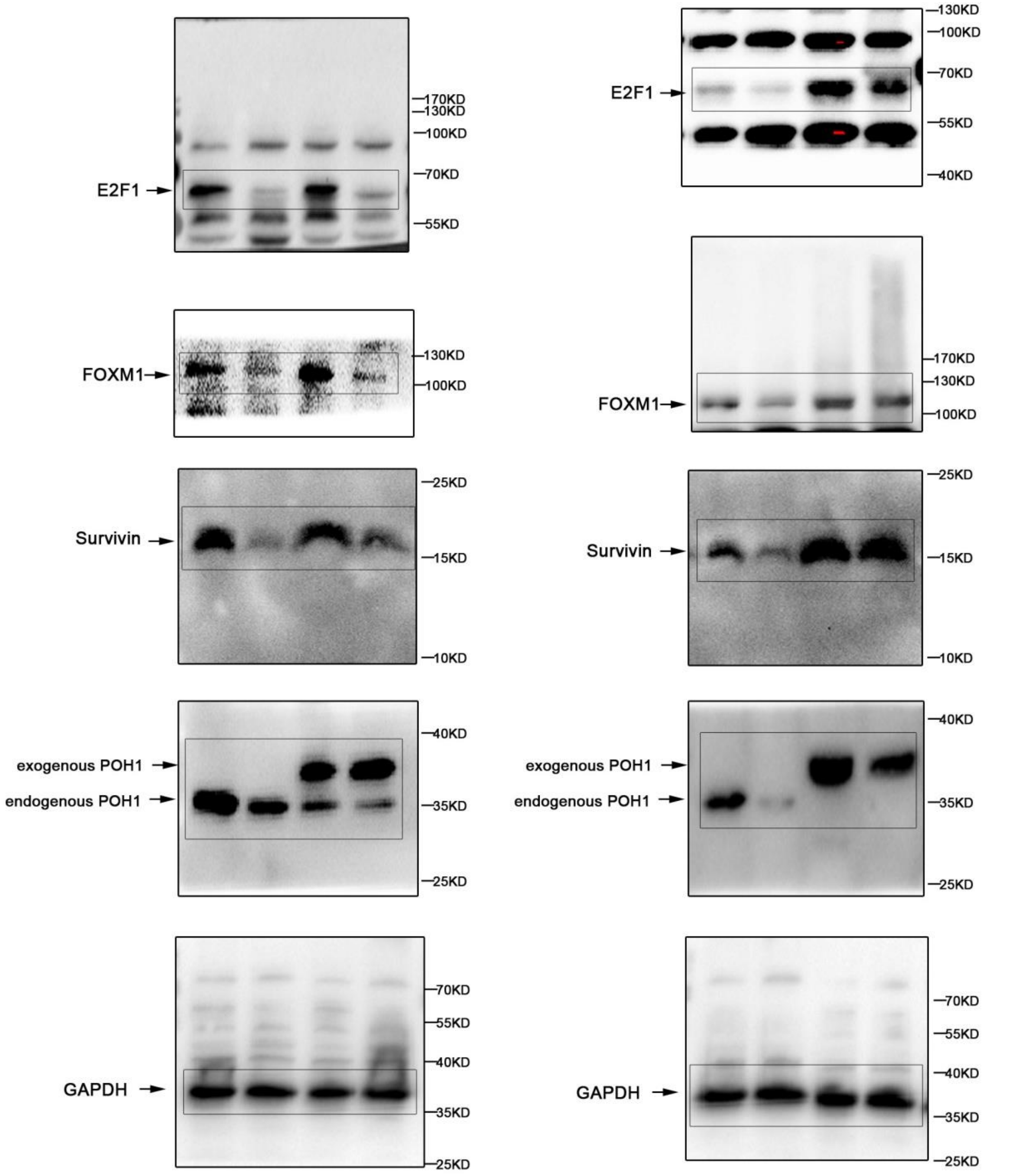

Supplementary Fig.17 continued

Figure 6.i

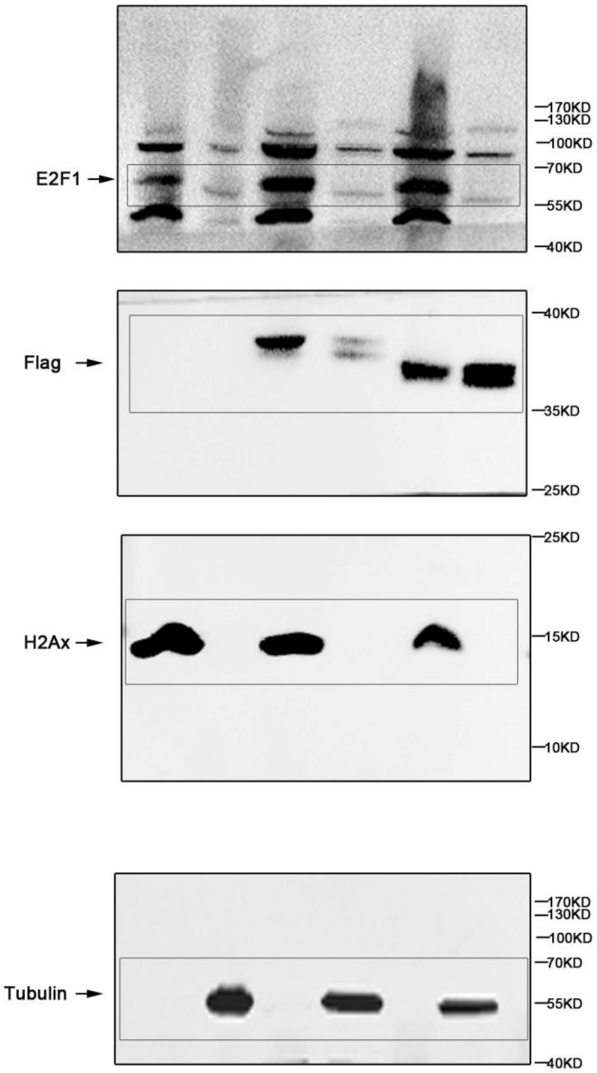

Supplementary Fig. 17 continued

Figure 7.a

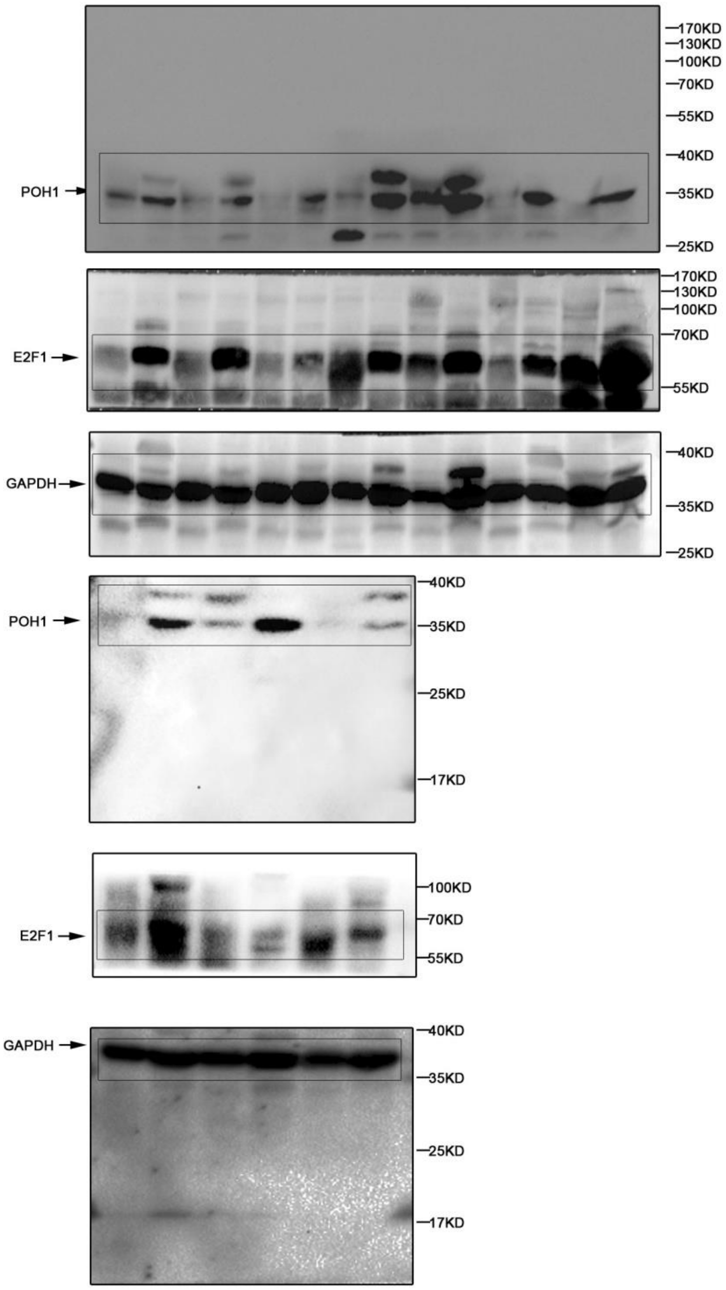

Supplementary Fig.17 continued

Supplementary Figure 1.a

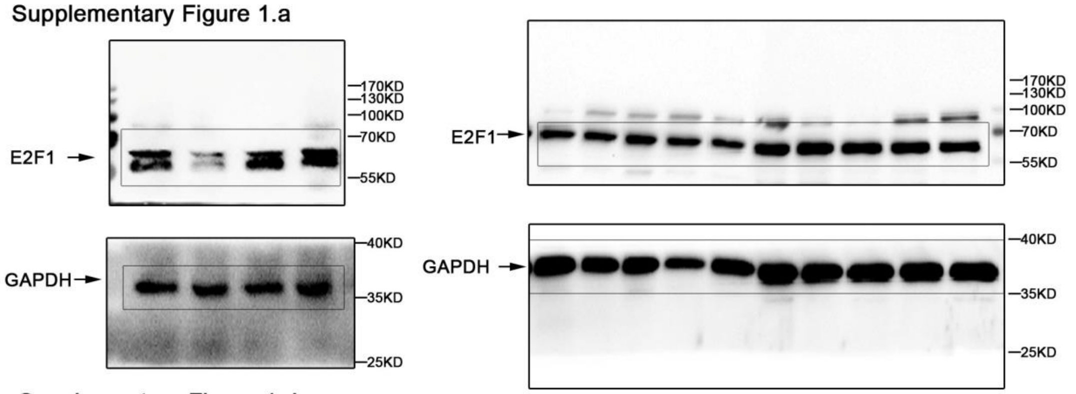

Supplementary Figure 1.d

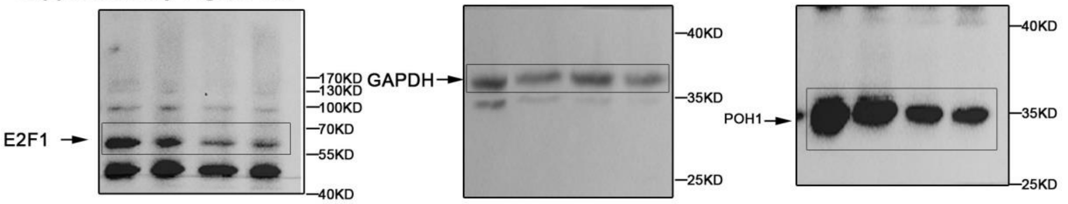

Supplementary Figure 1.e

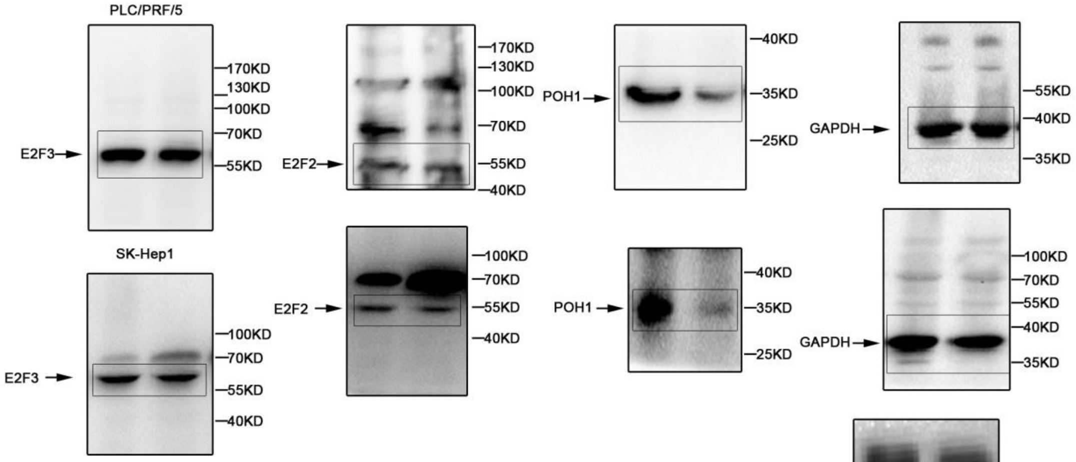

Supplementary Figure 1.i

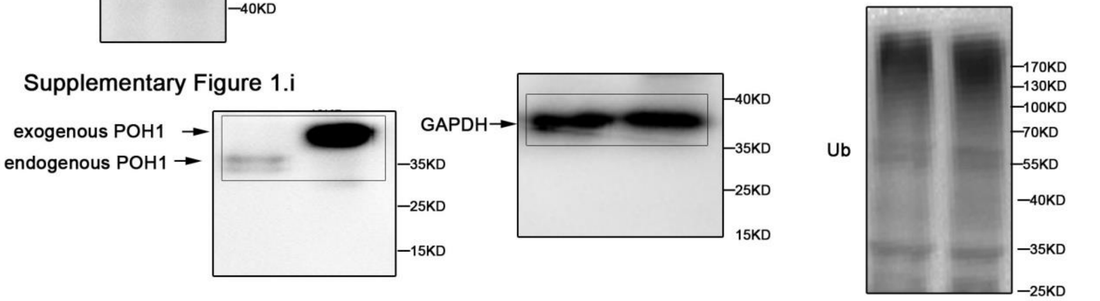

Supplementary Fig.17 continued

Supplementary Fig 2.a

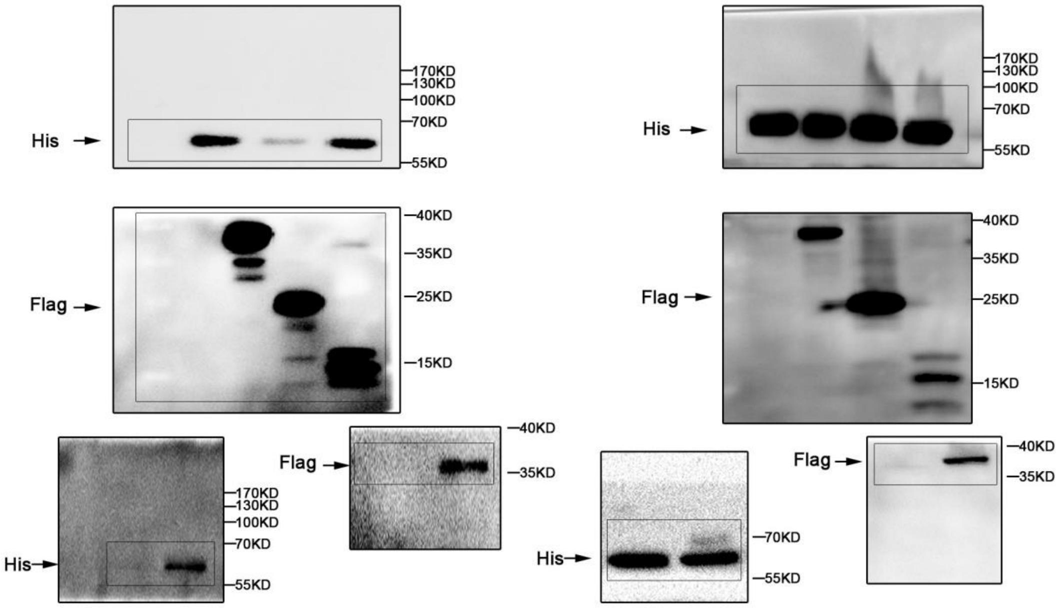

Supplementary Fig 2.b

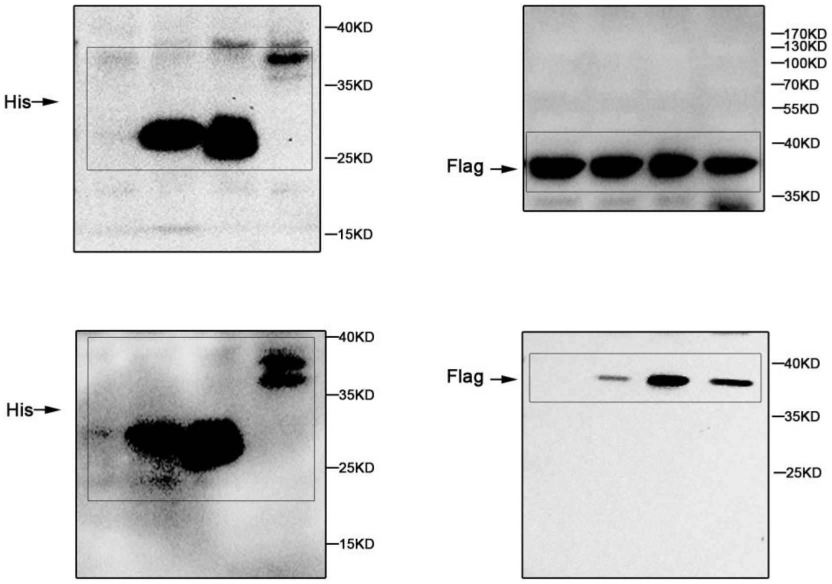

Supplementary Fig.17 continued

Supplementary Fig 3.c

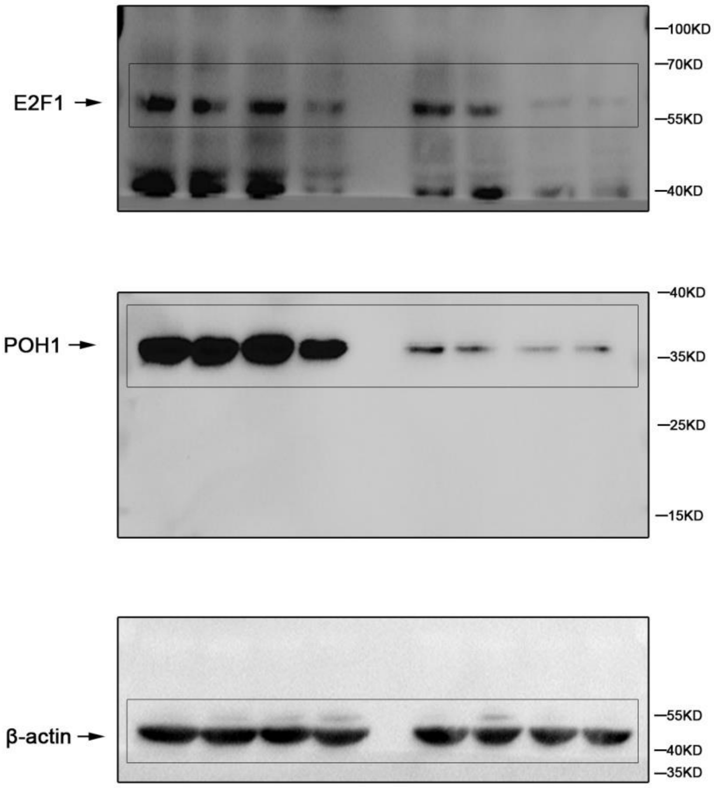

Supplementary Fig.17 continued

Supplementary Fig 4. a

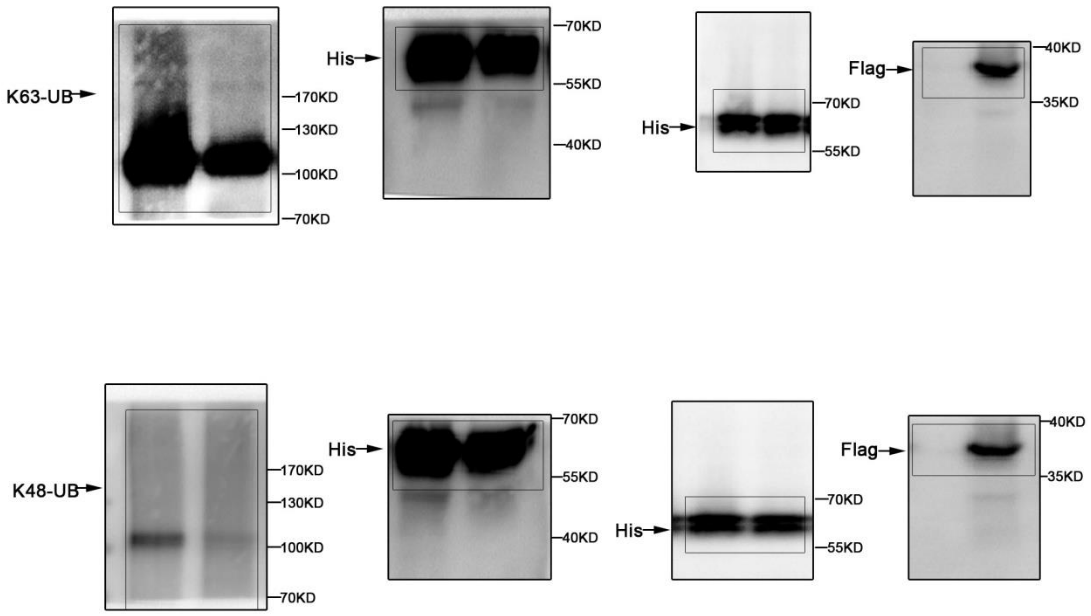

Supplementary Fig 4. b

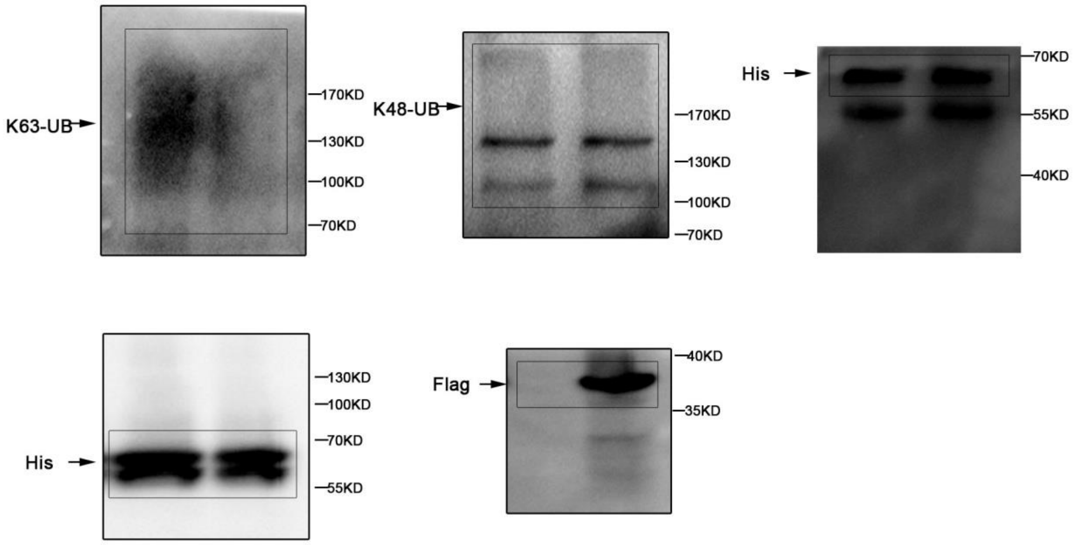

Supplementary Fig.17 continued

Supplementary Fig 5.a

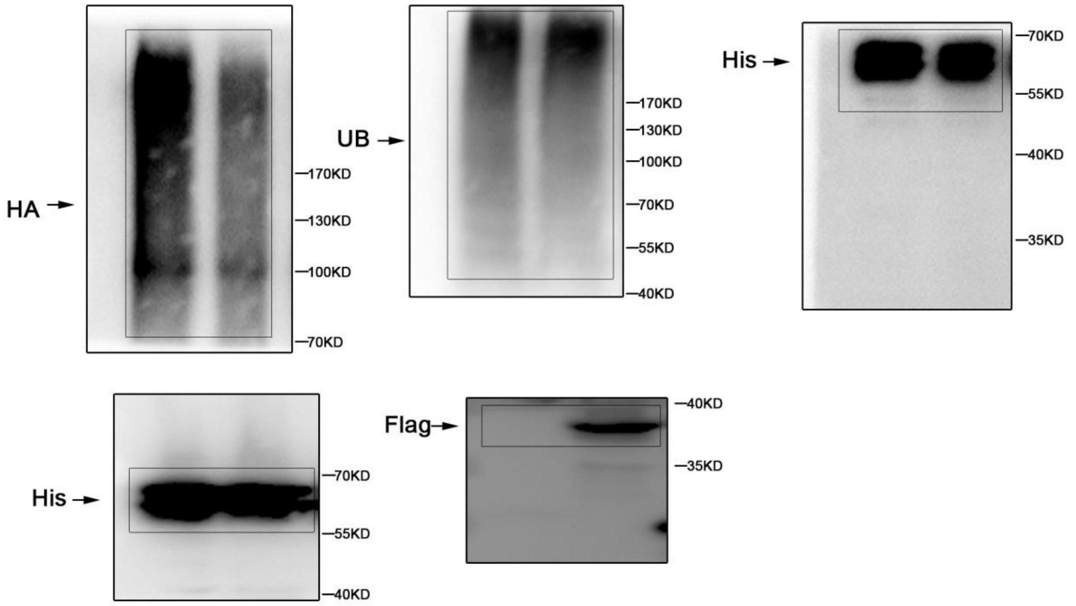

Supplementary Fig 5.b

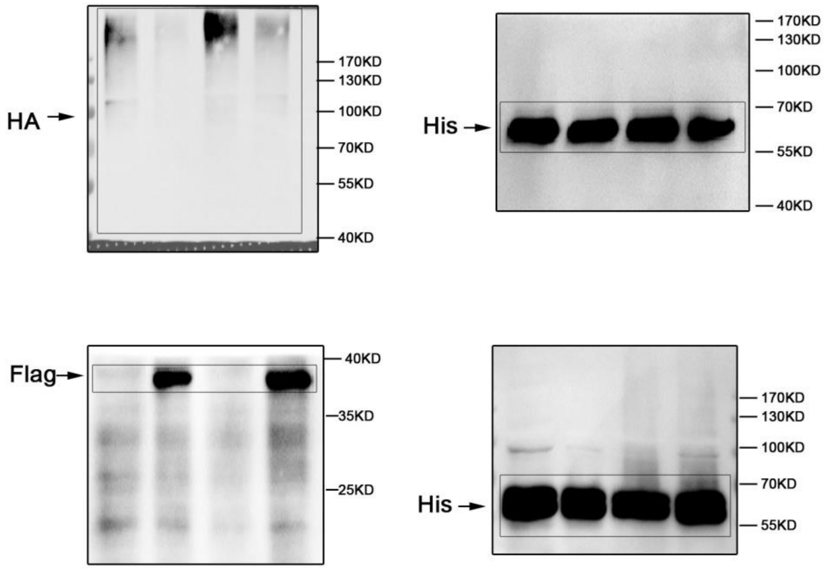

Supplementary Fig.17 continued

Supplementary Fig 6.h

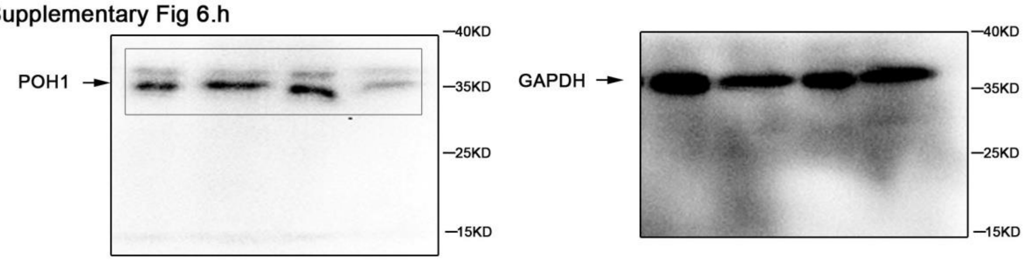

Supplementary Fig 10

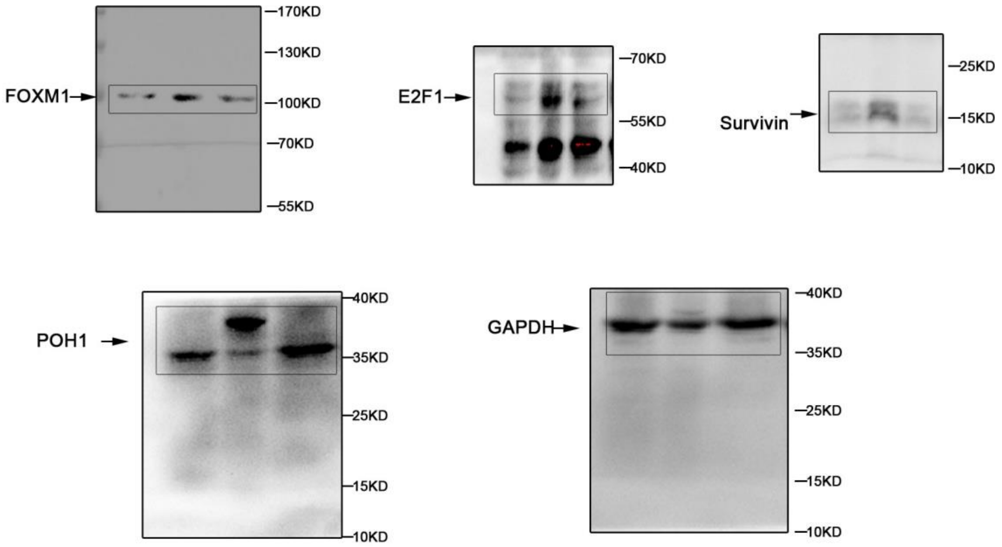

Supplementary Fig 11.a

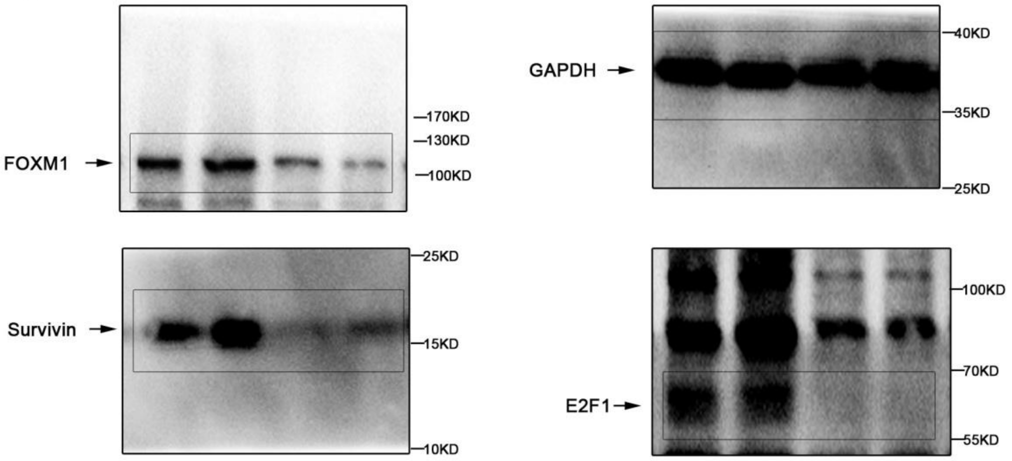

Supplementary Fig.17 continued

Supplementary Fig 13

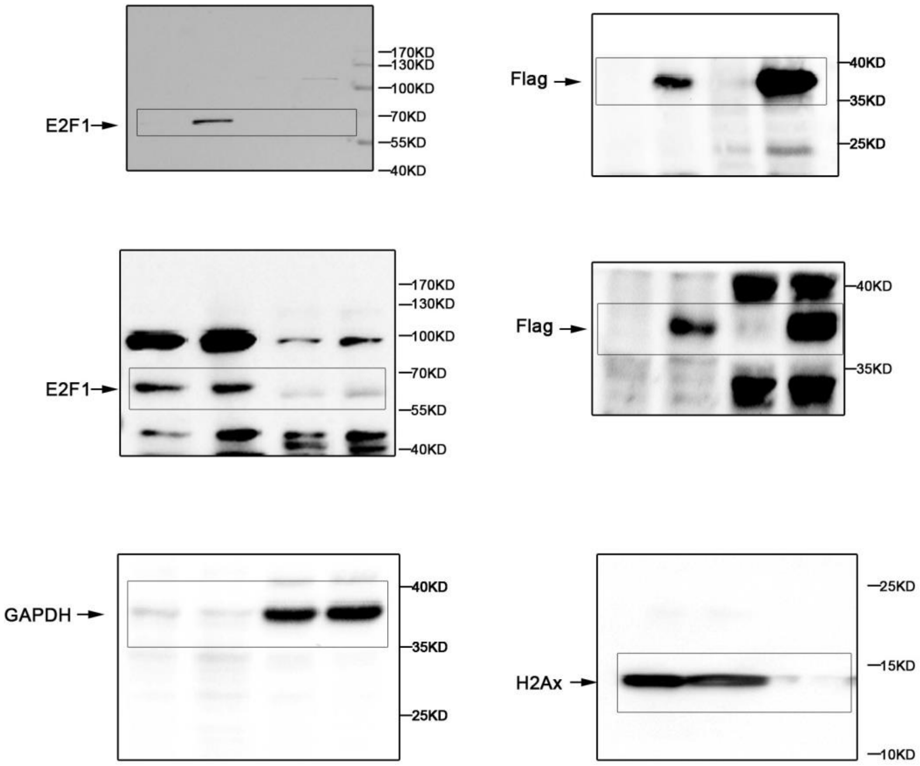

Supplementary Fig.17 continued

Supplementary Figure 17. Scans of uncropped blots of the figures.

Supplementary Table 1. The list of E2F1-regulated genes and the related publications

Genes	Publications
TP53BP2	Cell Death Differ. 2005 Apr;12(4):369-76; Cell Death Differ. 2005 Apr;12(4):377-83.
JMY	Cell Death Differ. 2005 Apr;12(4):377-83.
PPP1R13B	Cell Death Differ. 2005 Apr;12(4):369-76; Cell Death Differ. 2005 Apr;12(4):377-83.
MSH2	Nat Cell Biol. 2005 Feb;7(2):137-47. Epub 2004 Dec 26; Genes Dev. 2002 Jan 15;16(2):245-56.
Survivin	J Biol Chem. 2004 Sep 24;279(39):40511-20; J Biol Chem. 2013 Apr 26;288(17):12032-41.
RAD54L	Genes Dev. 2002 Jan 15;16(2):245-56.
MAL2	Cancer Cell. 2008 Jan;13(1):11-22.
MYBL2	Genes Dev. 2000 Apr 1;14(7):804-16.
STMN1	Ann Surg Oncol. 2013 Nov;20(12):4041-54.
CDC6	Genes Dev. 2000 Apr 1;14(7):804-16.
FOXM1	Mol Cancer Res. 2012 Sep;10(9):1189-202; Mol Cancer Ther. 2011 Jun;10(6):1046-58.
CDK1	Genes Dev. 2000 Apr 1;14(7):804-16.
NUSAP1	Prostate. 2015 Apr 1;75(5):517-26.
CCNB1	Cancer Cell. 2008 Jan;13(1):11-22.
BARD1	Genes Dev. 2002 Jan 15;16(2):245-56.
CCNA2	Nature. 2004 Aug 12;430(7001):797-802; Dig Liver Dis. 2011 Dec;43(12):1006-14.
CDC25A	Genes Dev. 2000 Apr 1;14(7):804-16.
CDC20	Cancer Cell. 2008 Jan;13(1):11-22.
MMP9	Cancer Res. 2012 Jan 15;72(2):516-26; PLoS One. 2013 Sep 4;8(9):e73436.
CHEK1	Genes Dev. 2002 Jan 15;16(2):245-56.
MAD2L1	Genes Dev. 2002 Jan 15;16(2):245-56; Nature. 2004 Aug 12;430(7001):797-802.
LMNB1	Cancer Cell. 2008 Jan;13(1):11-22.
TP53INP1	Cell Death Differ. 2005 Apr;12(4):377-83.
SKP2	Oncogene. 2006 Apr 27;25(18):2615-27; Cancer Lett. 2013 Jun 1;333(1):124-32.
EGR1	Hepatology. 2014 Sep;60(3):919-30; Cancer Res. 2009 Mar 15;69(6):2324-31.
TOP2A	Cancer Cell. 2008 Jan;13(1):11-22.
KIF20A	Cancer Cell. 2008 Jan;13(1):11-22.
RBL1	Genes Dev. 2000 Apr 1;14(7):804-16.
PLK1	Exp Cell Res. 2013 Dec 10;319(20):3104-15.

FMR1	Cancer Cell. 2008 Jan;13(1):11-22.
IQGAP3	Cancer Cell. 2008 Jan;13(1):11-22.
PTTG1	Mol Endocrinol. 2009 Dec;23(12):2000-12.

Supplementary References

1. Fogal, V. *et al.* ASPP1 and ASPP2 are new transcriptional targets of E2F. *Cell death and differentiation* **12**, 369-376 (2005).
2. Hershko, T., Chaussepied, M., Oren, M. & Ginsberg, D. Novel link between E2F and p53: proapoptotic cofactors of p53 are transcriptionally upregulated by E2F. *Cell death and differentiation* **12**, 377-383 (2005).
3. Youn, C.K. *et al.* Bcl-2 expression suppresses mismatch repair activity through inhibition of E2F transcriptional activity. *Nature cell biology* **7**, 137-147 (2005).
4. Ren, B. *et al.* E2F integrates cell cycle progression with DNA repair, replication, and G(2)/M checkpoints. *Genes & development* **16**, 245-256 (2002).
5. Jiang, Y., Saavedra, H.I., Holloway, M.P., Leone, G. & Altura, R.A. Aberrant regulation of survivin by the RB/E2F family of proteins. *The Journal of biological chemistry* **279**, 40511-40520 (2004).
6. Kan, C.Y. *et al.* Up-regulation of survivin during immortalization of human myofibroblasts is linked to repression of tumor suppressor p16(INK4a) protein and confers resistance to oxidative stress. *The Journal of biological chemistry* **288**, 12032-12041 (2013).
7. Hallstrom, T.C., Mori, S. & Nevins, J.R. An E2F1-dependent gene expression program that determines the balance between proliferation and cell death. *Cancer cell* **13**, 11-22 (2008).
8. Takahashi, Y., Rayman, J.B. & Dynlacht, B.D. Analysis of promoter binding by the E2F and pRB families in vivo: distinct E2F proteins mediate activation and repression. *Genes & development* **14**, 804-816 (2000).
9. Chen, Y.L. *et al.* The E2F transcription factor 1 transactives stathmin 1 in hepatocellular carcinoma. *Annals of surgical oncology* **20**, 4041-4054 (2013).
10. de Olano, N. *et al.* The p38 MAPK-MK2 axis regulates E2F1 and FOXM1 expression after epirubicin treatment. *Molecular cancer research : MCR* **10**, 1189-1202 (2012).
11. Millour, J. *et al.* ATM and p53 regulate FOXM1 expression via E2F in breast cancer epirubicin treatment and resistance. *Molecular cancer therapeutics* **10**, 1046-1058 (2011).
12. Gordon, C.A., Gulzar, Z.G. & Brooks, J.D. NUSAP1 expression is upregulated by loss of RB1 in prostate cancer cells. *The Prostate* **75**, 517-526 (2015).
13. Hernando, E. *et al.* Rb inactivation promotes genomic instability by uncoupling cell cycle progression from mitotic control. *Nature* **430**, 797-802 (2004).
14. Farra, R. *et al.* Effects of E2F1-cyclin E1-E2 circuit down regulation in hepatocellular carcinoma cells. *Digestive and liver disease : official journal of the Italian Society of Gastroenterology and the Italian Association for the Study of the Liver* **43**, 1006-1014 (2011).
15. Johnson, J.L. *et al.* Regulation of matrix metalloproteinase genes by E2F transcription factors: Rb-Raf-1 interaction as a novel target for metastatic disease. *Cancer research* **72**, 516-526 (2012).
16. Ma, X. *et al.* Overexpression of E2F1 promotes tumor malignancy and correlates with TNM stages in clear cell renal cell carcinoma. *PloS one* **8**, e73436 (2013).
17. Zhang, L. & Wang, C. F-box protein Skp2: a novel transcriptional target of E2F. *Oncogene* **25**, 2615-2627 (2006).
18. Xu, F. *et al.* The oncoprotein HBXIP up-regulates Skp2 via activating transcription factor E2F1 to promote proliferation of breast cancer cells. *Cancer letters* **333**, 124-132 (2013).
19. Zhang, Y. *et al.* E2F1 is a novel fibrogenic gene that regulates cholestatic liver fibrosis through

the Egr-1/SHP/EID1 network. *Hepatology* **60**, 919-930 (2014).

20. Zheng, C. *et al.* E2F1 Induces tumor cell survival via nuclear factor-kappaB-dependent induction of EGR1 transcription in prostate cancer cells. *Cancer research* **69**, 2324-2331 (2009).

21. Zhou, Z. *et al.* p53 Suppresses E2F1-dependent PLK1 expression upon DNA damage by forming p53-E2F1-DNA complex. *Experimental cell research* **319**, 3104-3115 (2013).

22. Zhou, C., Wawrowsky, K., Bannykh, S., Gutman, S. & Melmed, S. E2F1 induces pituitary tumor transforming gene (PTTG1) expression in human pituitary tumors. *Molecular endocrinology* **23**, 2000-2012 (2009).
